# Mechanism of cystogenesis by Cd79a-driven, conditional mTOR activation in developing mouse nephrons

**DOI:** 10.1038/s41598-023-27766-2

**Published:** 2023-01-10

**Authors:** Linh Tran Nguyen Truc, Satoshi Matsuda, Akiko Takenouchi, Quynh Tran Thuy Huong, Yui Kotani, Tatsuhiko Miyazaki, Hiroaki Kanda, Katsuhiko Yoshizawa, Hiroyasu Tsukaguchi

**Affiliations:** 1 Division of Nephrology, Second Department of Internal Medicine, Institute of Biomedical Science, Kansai Medical University, 2-5-1 Shinmachi, Hirakata, Osaka 573-1191 Japan; 2 Department of Cell Signaling, Institute of Biomedical Science, Kansai Medical University, 2-5-1 Shinmachi, Hirakata, Osaka 573-1010 Japan; 3 Department of Innovative Food Sciences, School of Food Sciences and Nutrition, Mukogawa Women’s University, Nishinomiya, Hyogo Japan; 4 Department of Diagnostic Pathology, Gifu University Hospital, Gifu, Japan; 5 Department of Pathology, Saitama Cancer Center, Saitama, Japan; 6 Department of Biological Science, Graduate School of Humanities and Sciences, Nara Women’s University, Nara, Japan

**Keywords:** Disease model, Polycystic kidney disease

## Abstract

Polycystic kidney disease (PKD) is a common genetic disorder arising from developmental and postnatal processes. Defects in primary cilia and their signaling (eg, mTOR) underlie the pathogenesis. However, how mTOR regulates tubular integrity remains unclear. The paucity of faithful models has limited our understanding of pathogenesis and, therefore, the refinement of therapeutic targets. To understand the role of mTOR in early cystogenesis, we studied an in-house mouse model, *Cd79a-Cre;Tsc1ff.* (Cd79a-Tsc1 KO hereafter), recapitulating human autosomal-dominant PKD histology. Cre-mediated Tsc1 depletion driven by the promoter for Cd79a, a known B-cell receptor, activated mTORC1 exclusively along the distal nephron from embryonic day 16 onward. Cysts appeared in the distal nephron at 1 weeks of age and mice developed definite PKD by 4 weeks. Cd79a-Tsc1 KO tubule cells proliferated at a rate comparable to controls after birth but continued to divide even after postnatal day 14 when tubulogenesis is normally completed. Apoptosis occurred only after 9 weeks. During postnatal days 7–11, pre-cystic Cd79a-Tsc1 KO tubule cells showed cilia elongation, aberrant cell intercalation, and mitotic division, suggesting that defective cell planar polarity (PCP) may underlie cystogenesis. mTORC1 was activated in a portion of cyst-lining cells and occasionally even when Tsc1 was not depleted, implying a non-autonomous mechanism. Our results indicate that mTORC1 overactivation in developing distal tubules impairs their postnatal narrowing by disrupting morphogenesis, which orients an actively proliferating cell toward the elongating axis. The interplay between mTOR and cilium signaling, which coordinate cell proliferation with PCP, may be essential for cystogenesis.

## Introduction

Polycystic kidney disease (PKD) is a common genetic disorder affecting 1 in 1000 individuals^1,2^. The vast majority of PKD subtypes are autosomal-dominant (ADPKD), and are caused by a two-hit, loss of function in either the polycystin *PKD1* (85%) or *PKD2* (15%) genes^3^. Individuals with ADPKD usually exhibit renal dysfunction by 40 years of age, and 50% of patients progress to ESRD (end-stage renal disease) by the age of 60^4^. Considerable intra- or interfamilial phenotypic variability suggests the presence of genetic modifiers^5^. A severe fetal or in utero ADPKD phenotype occurs in 1–2% of those affected^6,7^. For example, this occurs in cases of a contiguous deletion of the *PKD1* and *TSC2* genes, where the loss of *TSC2* exacerbates the PKD1 depletion phenotype. Autosomal recessive PKD, where the *PKHD* gene is mutated, is another early-onset subtype of PKD. All these cystogenic genes converge on the cilia of tubules, leading to the concept of PKD being a “ciliopathy.”

The genes *TSC1* and *TSC2* encode tumor suppressors and their loss of function leads to the inherited hamartomas syndrome tuberous sclerosis complex (TSC). Renal lesions consist of angiomyolipomas, renal cysts, and renal cell carcinomas. Tsc1/2 regulates the mammalian target of rapamycin (mTOR) pathway that mediates various cellular processes, including cell size, proliferation, survival, and protein synthesis^8,9^. mTOR signaling occurs via two multiprotein complexes, namely mTOR complex 1 (mTORC1) and 2 (mTORC2). mTORC1 comprises the core molecule Raptor and interacts with the ribosomal protein S6 kinase (S6K1) and other downstream effectors. TSC1 is localized to the basal body of cilia and interacts with PKD1^10,11^. Tsc1/2 and PKD1/2 mutants share some features, but also show relevant differences^8,12,13^. Increased mTOR signaling is observed in cysts in both mouse and human ADPKD^14,15^. These observations point to the relevance of the mTOR pathway in cystogenesis^11,16^.

Given that cyst formation early in life significantly contributes to cystic burden in adulthood, understanding initial cystogenesis events can have profound therapeutic benefits. In rodent models, the distal tubules are most susceptible to cystogenesis^17–20^. The most severe cysts manifest in mice model when a loss of the PKD-causing genes occurs during postnatal days (P) 0 to P13^20–22^. This strict time-dependency of cystogenic competence reflects a sharp developmental switch of proliferation and gene expression, which is important for segment-specific morphogenesis^20,21,23^. Multiple cellular processes have been implicated in the pathogenesis of PKD. Most are cilia-related functions, such as proliferation, apoptosis, and planar cell polarity (PCP)^24,25^. PCP signaling regulates tubulogenesis through the coordination of convergent extension (CE) and oriented cell division (OCD), both of which are mechanisms that shape developing renal tubules^26,27^. CE is a directed intercalation process of tubular cells that changes the direction of their long axis from perpendicular to parallel relative to the tubular axis^27–30^. OCD aligns the longitudinal tubular axis around the time of birth, thereby ensuring postnatal tubule narrowing and lengthening^28,29^. However, how CE and OCD contribute to the PKD phenotype and how they are functionally linked to the cilium is still under debate^30,31^.

Here, we studied our in-house Cre line *Cd79a-Cre;Tsc1ff.* (Cd79a-Tsc1 KO) to evaluate how mTOR signals are involved in early cystogenesis. Cd79a is a B-cell receptor and its promoter has been used to drive Cre-mediated deletion in the B-cell lineage^32^. We and others serendipitously found that Cd79a-Tsc1 KO mice display a PKD phenotype, but the underlying mechanisms are still unclear. Unlike a previous Tsc1 KO model^33^, the Cd79a-Tsc1 KO is unique in that aberrant mTOR activation in the ureteric buds starts no earlier than embryonic day (E) 16. Such conditional mTOR activation leads to aberrant cell proliferation that is restricted to developing distal tubules, which undergo coordinated narrowing and elongation. Moreover, pre-cystic Cd79a-Tsc1 KO tubule cells display abnormal cilia elongation as well as defective PCP, resulting in alterations in both CE and OCD. All of these mechanisms may relate and contribute to cyst formation.

## Results

### Tsc1 inactivation by Cd79a-Cre causes PKD

**Figure 1:**
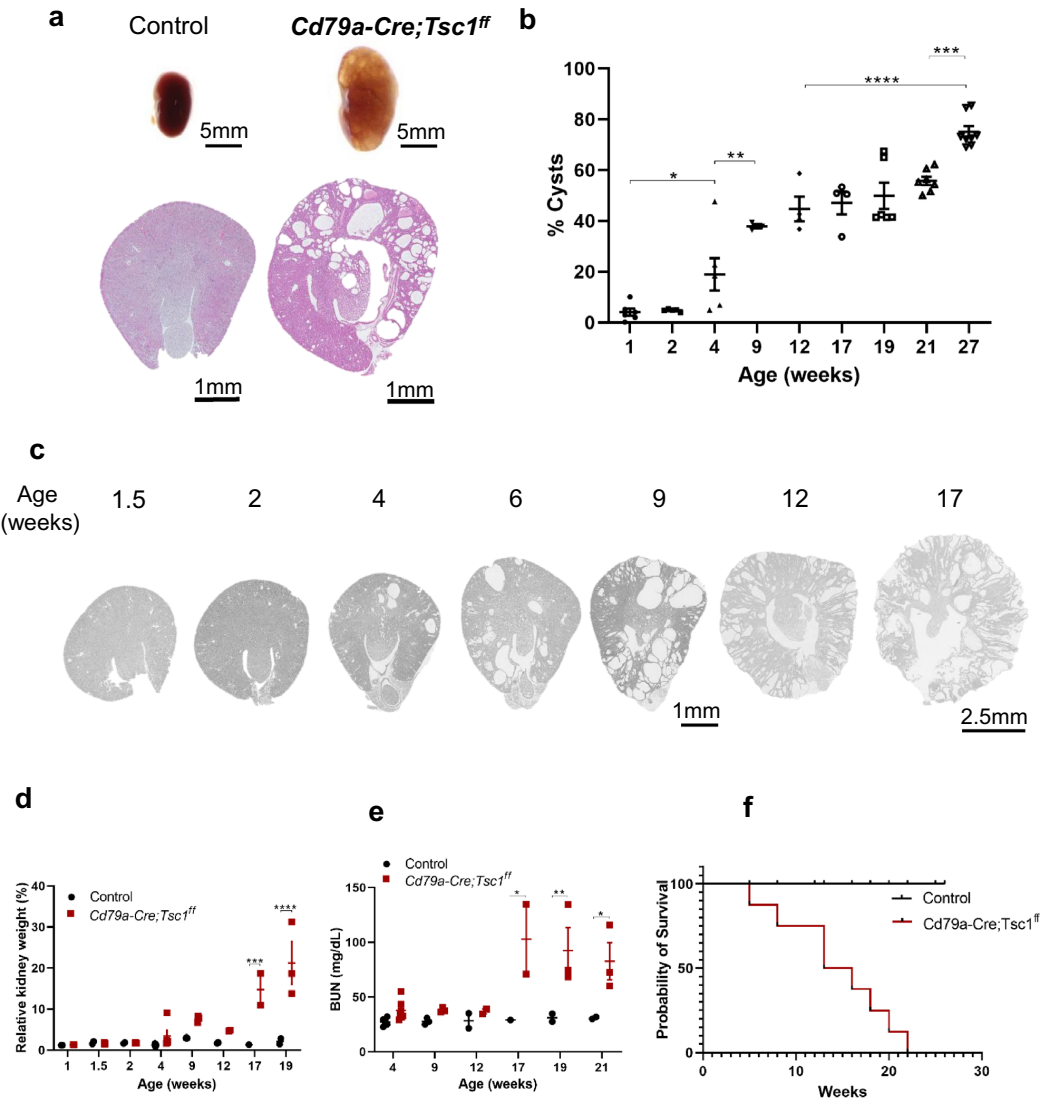
Cyst progression in Cd79a-Tsc1 knockout (KO) mouse kidneys. (**a**) Gross appearance of a cystic kidney. Sections at 4 weeks of age by hematoxylin and eosin stain. (**b**, **c**) Time-course progression of renal cysts. A series of representative images from the age of 1.5 to 17 weeks and the corresponding cystic indices are shown. The cyst index is given by a fraction of cystic lesions in whole kidney area. (**d**) Progressive enlargement of Cd79a-Tsc1 KO kidneys. A dramatic increase in kidney weight is observed after 4 weeks. The ratio of total kidney weight to body weight is indicated as a percentage. (**e**) Changes in renal function. Blood urea nitrogen (BUN, mg/dL) levels steeply rose after 12 weeks in Cd79a-Tsc1 KO mice compared with controls. (**f**) Kaplan–Meier analysis shows reduced viability of C79a-Tsc1 KO mice after 20 weeks of age. Control n = 15, male n = 8, female n = 7. Values represent mean ± SEM, and statistical significance was determined by two-way ANOVA. *P < 0.05, **P < 0.01, ***P < 0.001 and ****P < 0.0001.

Cd79a-Cre–mediated, conditional Tsc1 KO mice (CD79a-Tsc1 KO: genotype *Cd79a-Cre;Tsc1ff.*) developed an overt PKD phenotype postnatally by the age of 4 weeks (Fig. 1a). The renal phenotype was fully penetrant and always manifested bilaterally, and cysts gradually increased in size and number as the mouse aged (Fig. 1b,c). Macroscopic inspection revealed no extra-renal phenotypes besides atrophy of the spleen. However, a detailed analysis of the immune phenotypes demonstrated that, in bone marrow of Cd79a-Tsc1-KO mice, a proportion of immature B cells is significantly reduced, while those of pro-B cells and pre-B cells remain intact (Supplementary Fig. S1a). Moreover, in the spleen, a proportion of follicular B (FO-B) cells and marginal zone B (MZ-B) cells was distinctly decreased in Cd79a-Tsc1-KO mice, compared with those in control mice. The findings are in line with previous observations (Supplementary Fig S1b)^34,35^. Consistent with the nearly complete loss of MZ-B cells, serum IgM and IgA levels were reduced in Cd79a-Tsc1-KO mice (Supplementary Fig. S2a). Systematic histopathological examinations disclosed no infiltration of mononuclear cells or inflammatory foci in whole-body organs, with the exception of occasional, focal inflammatory reactions seen in the heart and peritoneum (Supplementary Fig. S3, 4). Homozygous *Cd79a-Cre;Tsc1ff.* mice were born at the expected Mendelian ratios and had a normal appearance. No PKD phenotypes were observed in heterozygous *Cd79a-Cre;Tsc1f.*^*/*+^ mice, indicating the null allele is recessive. Kidney weights of the Cd79a-Tsc1 KO mice gradually increased starting at 4 weeks of age (Fig. 1d). The survival rate for Cd79a-Tsc1 KO mice decreased with age in proportion to the BUN (blood urea nitrogen) rise starting at 12 weeks of age (Fig. 1e). Most mice died after 20 weeks of age, and there was no gender difference in survival (Fig. 1f).

**Figure 2:**
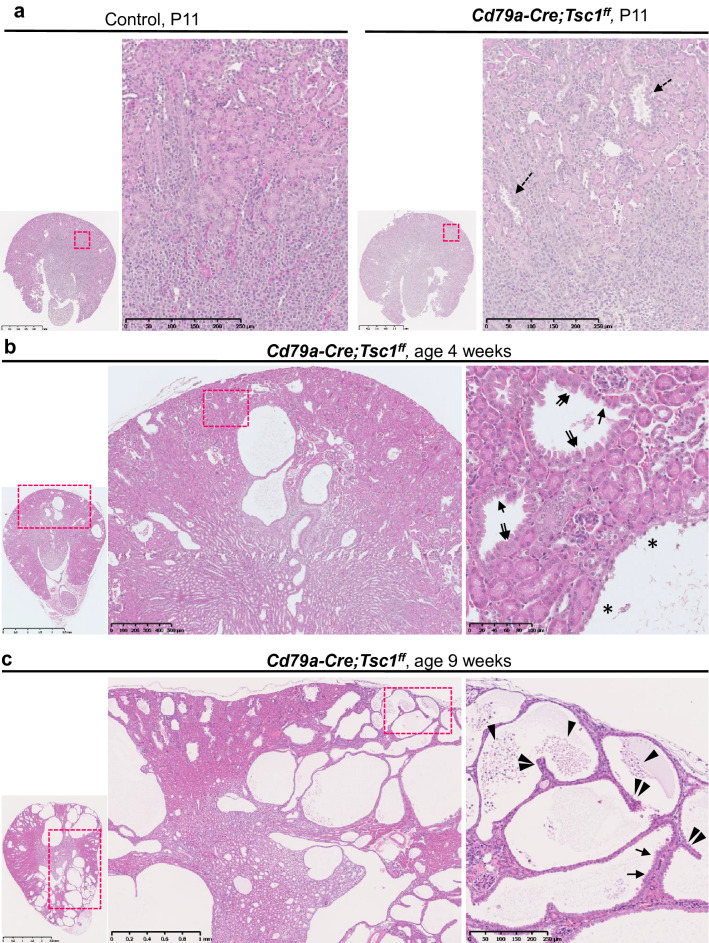
Features of cystogenesis in Cd79a-Tsc1 knockout (KO) mouse kidneys. Representative histology sections of Cd79a-Tsc1 KO kidneys are shown at postnatal day (P) 11 (**a**), 4 weeks (**b**), and 9 weeks (**c**). At P11, Cd79a-Tsc1 KO tubules exhibited cysts, which initially emerged in the peripheral cortex (dashed arrows) and expanded to the medulla at 4 weeks of age. The early cysts at 4 weeks were lined with epithelial cells and had an array of morphologic subtypes: low cuboidal (arrows), flattened (asterisks), and tall columnar/hypertrophic with karyocytomegaly (double arrows). In the expanding cysts at the later stage of 9 weeks, a fraction of the cells detached and exfoliated into the tubular lumens (arrowheads). An intense focal proliferation was occasionally observed (micropapillary lesion, double arrowheads). Dashed box areas are magnified.

Light microscopic examination of Cd79a-Tsc1 KO kidneys showed that cysts become discernable as early as postnatal week 1, while no cysts were observed prenatally. Microscopic cysts typically appeared from the peripheral cortex, then expanded to the medulla and were grossly enlarged by 4 weeks. There were several types of early (4-week) cysts, based on what they were lined by: cuboidal, hypertrophic cells with karyocytomegaly; flat, dedifferentiated epithelium; and/or partial multi-layer columnar epithelia with intraluminal cell exfoliation in expanding cysts (Fig. 2a–c). Notably, after 4 weeks of age, cysts began to appear as more tumorous, papillary, or solid mass lesions (Fig. 2c).

**Figure 3:**
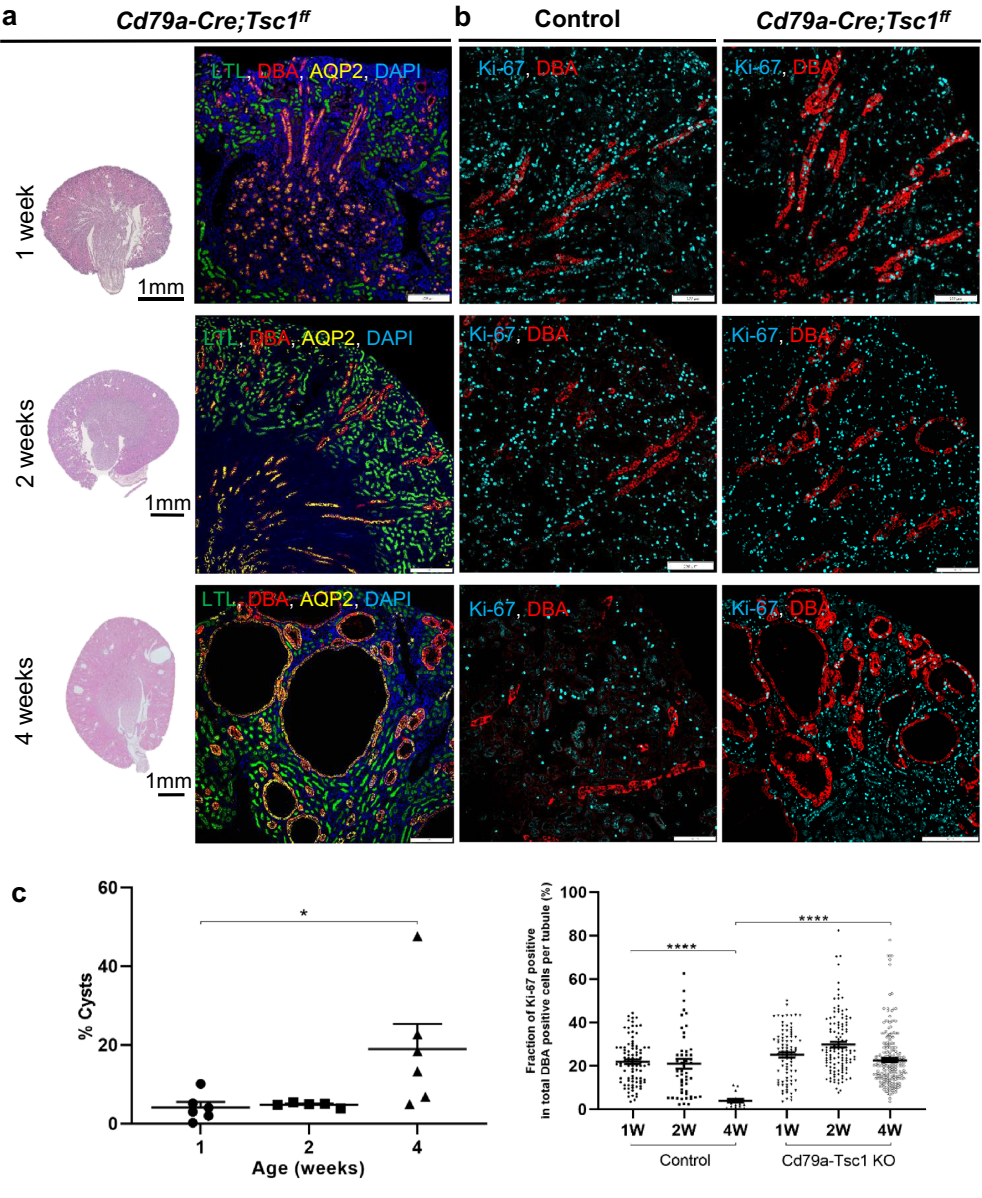
Renal phenotypes at early postnatal stages in Cd79a-Tsc1 knockout (KO) mice. (**a**) Kidney sections (hematoxylin and eosin stain) and histology. Postnatal tubules at 1 to 4 weeks of age are stained by specific markers: LTL (proximal tubule), DBA (distal tubules to collecting duct), and AQP2 (collecting duct). Cysts are visible as early as 2 weeks of age mainly from the distal tubules to the collecting ducts in the outer cortex. (**b**) Comparison of proliferation activity between control and Cd79a-Tsc1 KO kidneys. Sections are stained by Ki-67. (**c**) Quantitative analysis. Cyst expansion is evaluated by the cystic index (%). Cysts grow remarkably after 2 weeks of age. Cell proliferation is assessed by counting the fraction of the pre-cystic and cystic tubule lining epithelial cells, in which the nuclei are costained with DBA and Ki-67 in the total number of DBA positive cells in each tubular. Proliferation of Cd79a-Tsc1 KO tubule cells persists even after 2 weeks of age, the time point of the developmental switch when proliferation abruptly ceases in control mice. Values represent mean ± SEM and statistical significance was determined by one-way ANOVA. *P < 0.05 and ****P < 0.0001.

We next stained cysts with tubular markers. From P11 to 9 weeks of age, most cysts were positive for DBA and AQP2, indicating that they originated mainly from the distal tubules and collecting ducts (Fig. 3a, Supplementary Fig. S5). At P11 in Cd79a-Tsc1 KO kidneys, AQP2 was expressed exclusively on the apical surface of the pre-cysts and cystic tubules. As the mice aged, a fraction of the cyst-lining cells were not positive for any tubular markers (Supplementary Fig. S5). At 4 weeks of age, AQP2 signal was also detected in the basolateral surface of large cysts, suggesting a loss of epithelial polarity.

### Cd79a-Cre activity in developing nephrons

We suspected that the PKD phenotype in Cd79a-Tsc1 KO mice arose from the unexpected, ectopic expression of Cd79a in the kidney. Cd79a is physiologically abundant in early pre-B cells, but may also exist to a lesser degree in other tissues, including the kidney. The GUDMAP database revealed that Cd79a is expressed in developing kidneys as early as E11.5 and reaches a maximum expression at E15.5 (Supplementary Fig. S6). Quantitative reverse transcriptase (RT)-PCR with total RNA from whole wild-type embryos revealed that Cd79a transcripts are expressed starting at E11.5 and reach a maximum plateau around E15.5 to E18.5 (Supplementary Fig. S7), consistent with the GUDMAP profiles.

**Figure 4:**
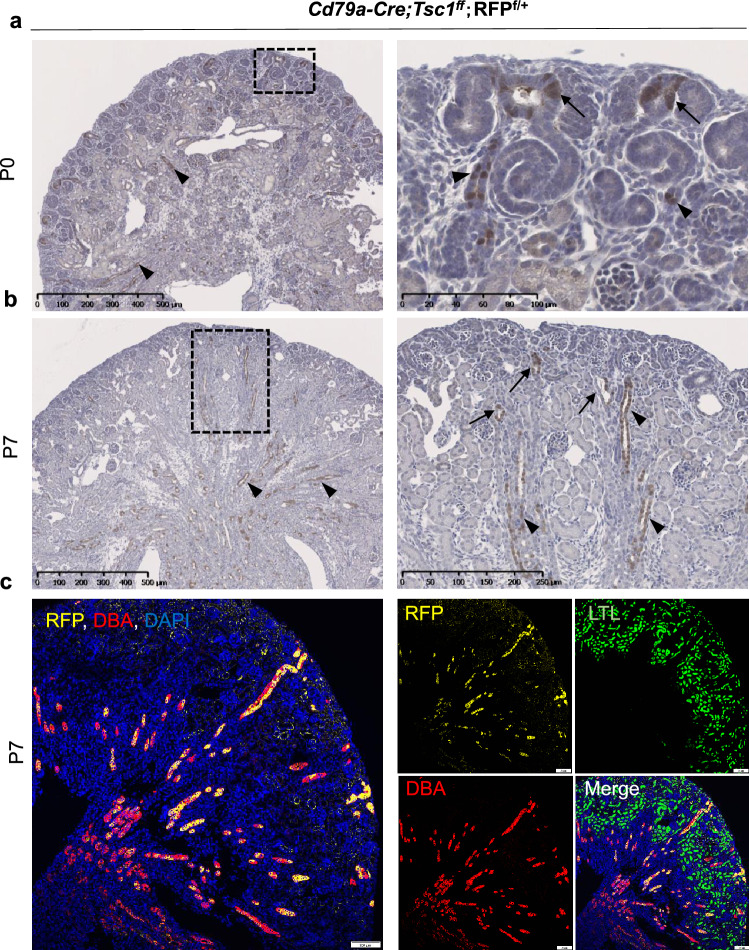
Localization of Cd79a-Cre activity in developing tubules of red fluorescent protein (RFP) reporter mice. Renal phenotypes of *Cd79a-Cre;Tsc1ff.;RFP*^*f/*+^ mice are shown. The RFP reporter traces cell lineage, and the Cd79a promoter drives Cre recombination. (**a**) Immunohistochemistry staining of kidney sections at postnatal day (P) 0, the RFP signal is found along the ureteric bud tip and stalk in the superficial nephrogenic cortex (arrows) and in the developing distal tubules of the medulla (arrowheads). (**b**) At P7, RFP signals extend over the medulla along the collecting ducts (arrowheads), in addition to being expressed in the distal convoluted tubules of the superficial cortex (arrows). (**c**) Immunofluorescence staining of kidney sections at P7 with an anti-RPF antibody as well as markers for proximal tubules (LTL, green) and distal tubules and collecting ducts (DBA, red). RFP signals mostly colocalized with DBA, indicating that the Cd79a promoter is active in ureteric buds and their progenitors in developing distal nephrons. Inset: Dashed box areas are magnified.

Immunohistochemistry failed to detect Cd79a around perinatal nephrons (data not shown). We thus investigated Cd79a-induced red fluorescent protein (RFP) reporter expression in *Cd79a-Cre;Tsc1ff.;RFP*^*f/*+^ mice. At birth (P0), RFP signals were abundant in ureteric buds and mature distal and collecting ducts, but were barely observed in mesenchymal cells, renal vesicles, and mature glomeruli (Fig. 4a,b). At P7, RFP signals colocalized mainly with DBA, a marker for distal tubules and collecting ducts, and did not colocalize with LTL, a marker for proximal tubules (Fig. 4c). Flow cytometric analysis of kidney lysate from *Cd79a-Cre;Tsc1f.*^*/*+^*;RFP*^*f/*+^ 3-month-old mice revealed that RFP-positive cells account for a small fraction (0.1%) of the epithelial-lineage subpopulation (Supplementary Fig. S8). Our results indicate that the Cd79a promoter is active in developing distal nephrons and is attenuated expression postnatally.

### mTOR signaling in Cd79a-Tsc1 KO kidneys

**Figure 5:**
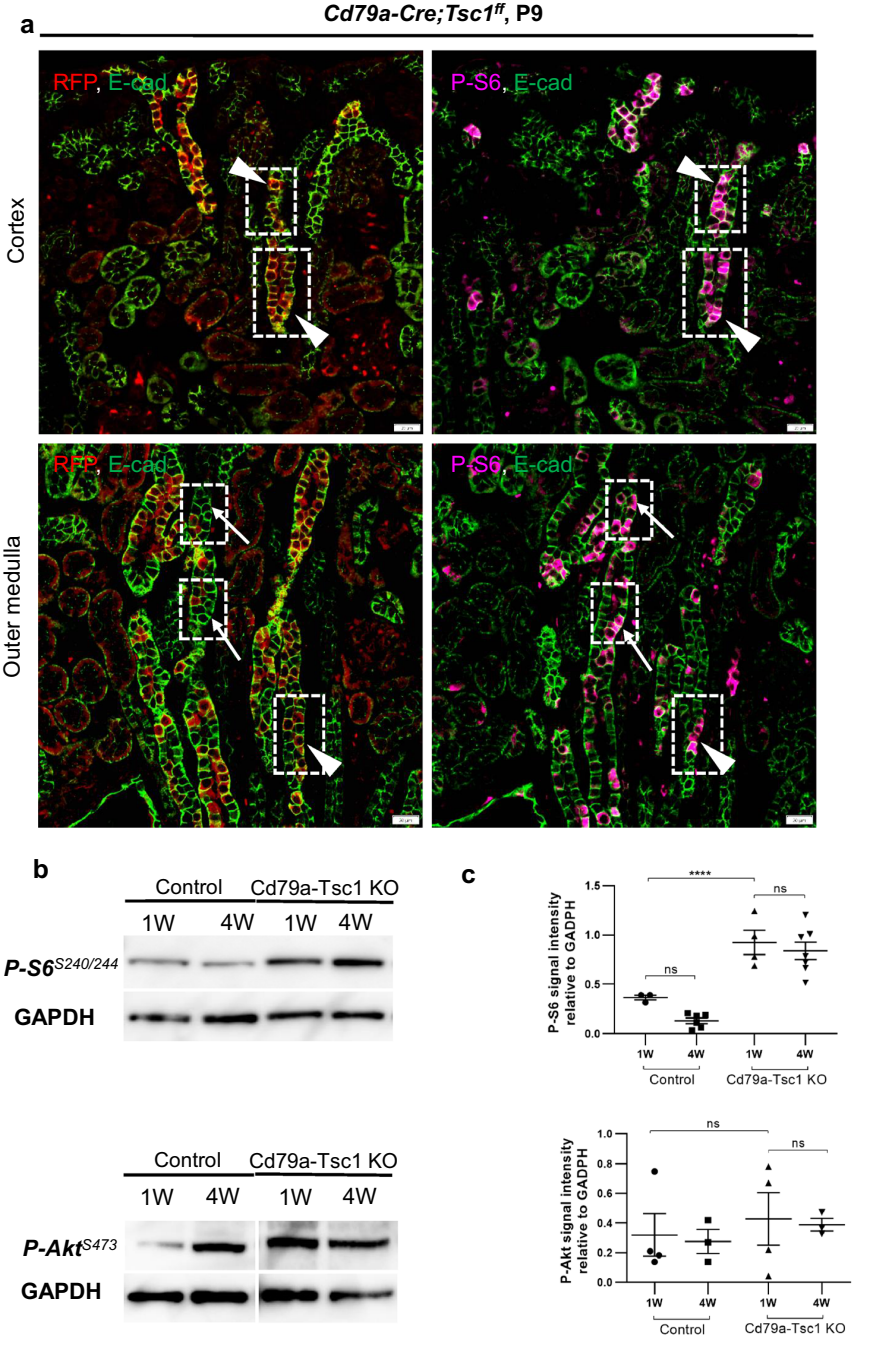
Autonomous and non-autonomous mTORC1 activation in pre-cystic tubules of Cd79a-Tsc1 knockout (KO) kidneys. (**a**) Two adjacent sections (3–5 µm) of *Cd79a-Cre;Tsc1ff.;RFP*^*f/*+^ mice at postnatal day (P) 9 are stained with antibodies against red fluorescent protein (RFP, a marker of Cd79a-Cre activity), phosphorylated (p)-S6 (an indicator of mTORC1 activation, magenta), and E-cadherin (E-cad, a marker of distal and collecting ducts, green). RFP signals are red in the cytoplasm, but are occasionally yellow when they merge with E-cadherin on the basal-lateral plasma membrane. Some epithelial cells coexpress p-S6 with RFP, while others are stained with the p-S6 only, suggesting that pre-cystic tubules are composed of two heterogeneous subpopulations of p-S6–positive cells: one undergoes Cre recombination (autonomous component, arrowheads) and the other does not (non-Cre recombination, non-autonomous component, arrows). (**b**) Immunoblot analysis of p-S6 (p-S6^S240/244^) and p-Akt (p-Akt^S473^). Analysis of whole kidney lysates indicates that p-S6 expression is enriched in Cd79a-Tsc1 KO kidneys compared to controls (upper panel). However, p-Akt abundance did not differ between the two groups (lower panel). The original gels was shown in Supplementary Fig. S12. (**c**) Quantification analysis. In Cd79a-Tsc1 KO kidneys, p-S6 and p-Akt expression levels at 1 week of age did not differ from those at 4 weeks. Data represent mean ± SEM and were statistically analyzed by one-way ANOVA. ****P < 0.0001.

We investigated mTORC1 signaling by immunostaining for the phosphorylated-S6 ribosomal protein (p-S6), an indicator of mTORC1 activation. In control kidneys, p-S6–positive cells were found mostly in LTL-positive, proximal tubules in the superficial cortex. Conversely, in Cd79a-Tsc1 KO mice, p-S6 signals were more frequent along the DBA-positive, pre-cystic and cystic distal nephrons (Supplementary Fig. S9). Notably, most, but not all, cells lining the pre- and early cysts were positive for p-S6, suggesting a mosaicism in tubule-component cells. We next examined whether p-S6 signals are linked to Tsc1 depletion by colocalizing p-S6 and the Cd79a-Cre–driven RFP reporter. In pre-cystic tubules of Cd79a-Tsc1 KO mice at P9, some p-S6–positive cells costained with RFP, while other p-S6 cells did not (Fig. 5a). The latter suggests non-autonomous mTORC1 activation. At 4 to 9 weeks of age, p-S6–positive staining was more frequent in cells lining the early growing cysts, but was conversely lower in flattened cells lining the massively expanding cysts (Supplementary Fig. S10). Staining for Akt phosphorylation (S473), an indicator of mTORC2 activation, was largely negative in Cd79a-Tsc1 KO tubules and it was indistinguishable from age-matched controls (data not shown).

**Figure 6:**
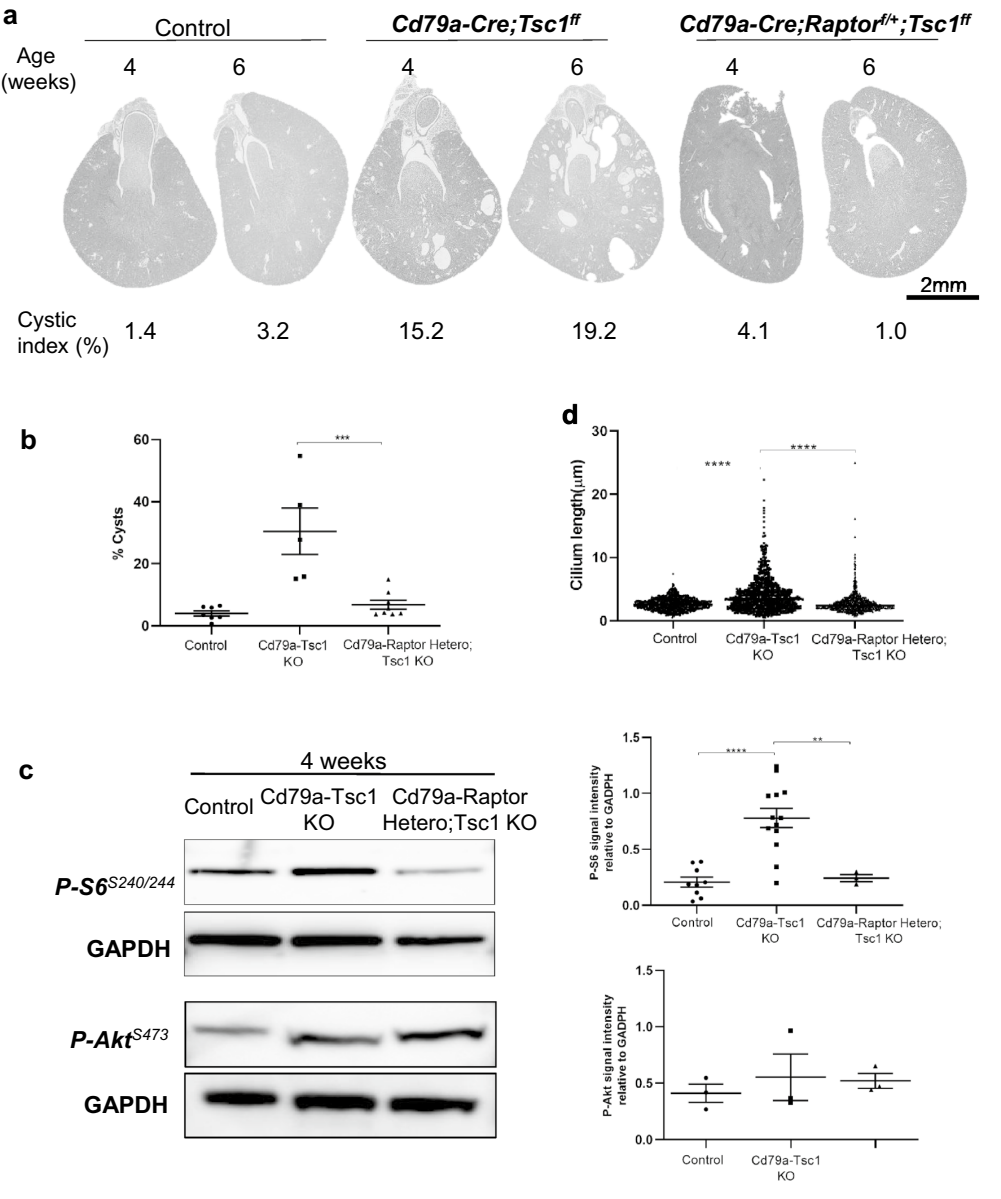
Heterozygous loss of Raptor suppresses cystogenesis in Cd79a-Tsc1 knockout (KO) kidneys. (**a**) Representative images of kidney sections from control, *Cd79a-Cre;Tsc1ff.*, and *Cd79a-Cre;Raptor*^*f/*+^*;Tsc1ff.* (Cd79a-Raptor Hetero;Tsc1 KO) mice at 4 and 6 weeks of age. (**b**) Comparison of cystic index at 4 and 6 weeks of age. Heterozygous loss of Raptor effectively suppresses cyst formation in Cd79a-Tsc1 KO kidneys. (**c**) Western blot analysis. Whole kidney lysates from each group were blotted with p-S6 and p-Akt antibodies. p-S6 expression was significantly reduced in Cd79a-Raptor Hetero;Tsc1 KO mice compared with Cd79a-Tsc1 KO mice. p-Akt expression did not differ among the three groups. The original gels was shown in Supplementary Fig. S13. (**d**) Quantification of cilia length (at 4 and 6 weeks) and mTOR signals. p-S6 and p-Akt signals were quantified from the immunoblot shown in (**c**). Concomitant heterozygous loss of Raptor reduced cilia length and p-S6 abundance in Cd79a-Tsc1 KO kidneys to a level comparable to control kidneys, suggesting that the cilia length defect is mediated by the mTORC1 complex. Values represent mean ± SEM and statistical significance was determined by one-way ANOVA. **P < 0.01, ***P < 0.001 and ****P < 0.0001.

Immunoblot analysis revealed that p-S6 was enriched in Cd79a-Tsc1 KO kidneys compared to controls (Fig. 5b). However, no differences in p-S6 levels were found across the early postnatal period from 1 to 4 weeks of age in Cd79a-Tsc1 KO tubules (Fig. 5c). The abundance of p-Akt in Cd79a-Tsc1 KO mice was indistinguishable from controls, and it did not change across postnatal weeks 1 to 4. When Raptor, a core component of the mTORC1 complex, was heterozygously deleted in Cd79a-Tsc1 KO kidneys (*Cd79a-Cre;Tsc1ff.;Raptor*^*f/*+^), cystogenesis was significantly suppressed at 4 and 6 weeks of age (Fig. 6a–c). Our data support the idea that the mTORC1 pathway plays a predominant role in the cystogenesis of our model.

### Proliferation and apoptosis in Cd79a-Tsc1 KO tubules

**Figure 7:**
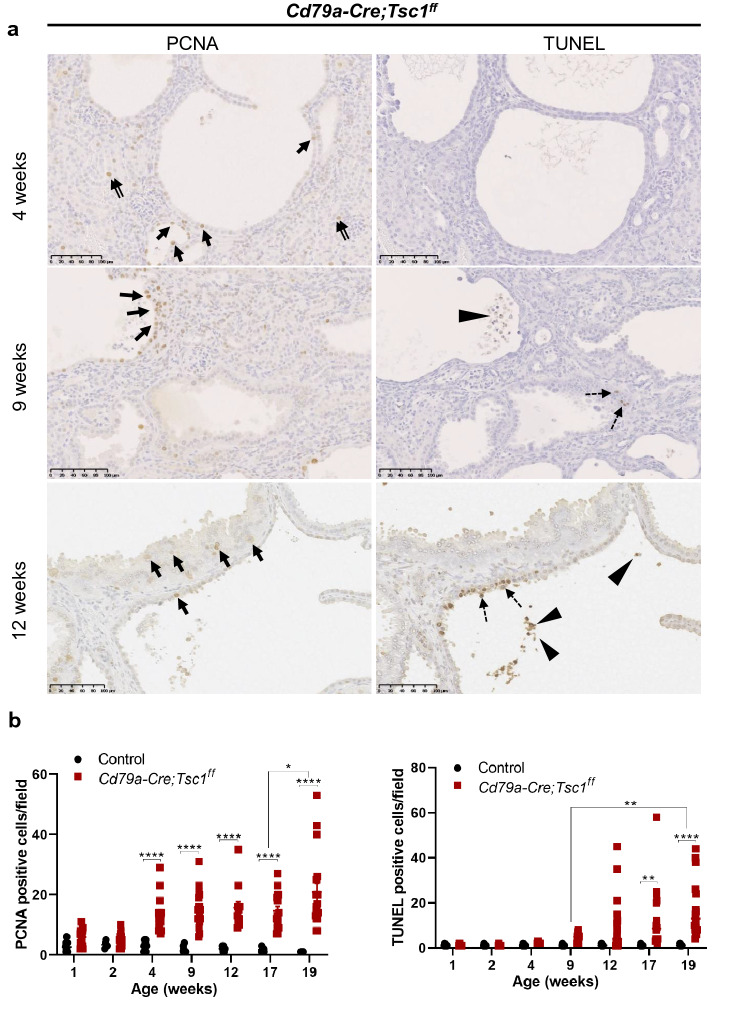
Proliferation and apoptosis in Cd79a-Tsc1 knockout (KO) kidneys. (**a**) Immunohistochemistry for proliferating cell nuclear antigen (PCNA) and terminal deoxynucleotidyl transferase dUTP nick end labeling (TUNEL) assay. Cd79a-Tsc1 KO kidneys were stained across developmental timepoints from postnatal week 1 to 19. In Cd79a-Tsc1 KO tubules at 4 weeks of age, PCNA-positive cells occasionally existed in both the cyst-lining (arrows) and non-cystic tubules (double arrows), while apoptosis was rarely observed. At 9 weeks, the number of PCNA-positive cells increased in the cyst-lining epithelia. In concordance with the increasing proliferation, apoptosis was more frequently observed in the cyst lining and interstitium. At later stages (12 weeks), apoptosis was seen focally in the cyst walls comprising multilayered, actively proliferating epithelial cells (dashed arrows) as well as in the cell debris that was exfoliated into the lumen (arrowheads). (**b**) Quantitative analysis. The frequencies of PCNA- and TUNEL-positive cells in tubules were greater in Cd79a-Tsc1 KO kidneys than in age-matched controls. After postnatal day 14, the enhanced proliferation in Cd79a-Tsc1 KO tubules became clearly discernible from controls (*P* < 0.0001). In parallel with the accelerated proliferation, apoptotic cells gradually increased with age and reached a significant difference compared to controls after 9 weeks. Data represent mean ± SEM and were statistically analyzed by two-way ANOVA. . *P < 0.05, **P < 0.01, and ****P < 0.0001.

We examined the proliferation of tubular cells. In control tubules, PCNA- or Ki-67–positive cells existed along developing tubules, but this expression abruptly declined over the first 2 postnatal weeks (Fig. 3b,c)^21^. By contrast, in Cd79a-Tsc1 KO tubules, cell proliferation was not appreciably different from controls during the first 2 postnatal weeks, and was even sustained after 2 weeks and further increased between the age of 4 to 19 weeks (Fig. 7a,b). Moreover, after 9 weeks, a robust focal proliferation was seen in tumorous lesions with papillary and/or solid masses.

We next evaluated apoptosis by the TUNEL assay. In postnatal kidneys of both control and Cd79a-Tsc1 KO mice, apoptotic cells occasionally exist in the medulla, but this expression abruptly declines over the first postnatal week^36^. In Cd79a-Tsc1 KO kidneys at 4 weeks of age, very few apoptotic nuclei were seen in the cyst lining and interstitium. At 9 weeks, apoptotic cells increasingly appeared, particularly in the multilayered, tumorous legions undergoing active proliferation, and in the luminal cell debris resulting from excessive exfoliation (Fig. 7a). Quantitative analysis revealed that apoptosis increased in step with cell proliferation activity (Fig. 7b). Our observations indicate that proliferation may trigger cystogenesis at earlier stages, while apoptosis becomes apparent only at later stages in response to overproliferation.

### Cilia morphology in Cd79a-Tsc1 KO tubules

**Figure 8:**
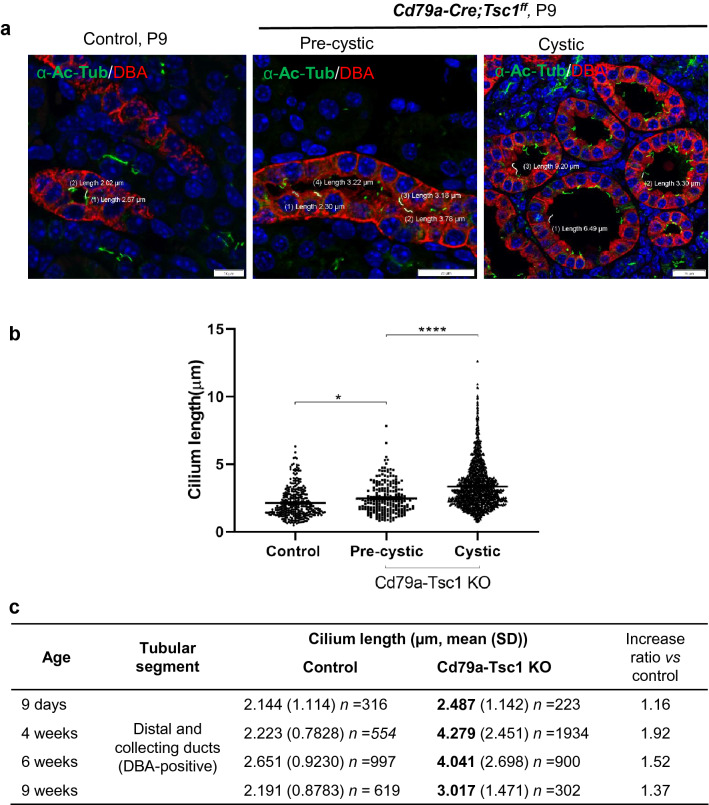
Morphologic changes of primary cilia during cyst progression. (**a**) Kidney sections of Cd79a-Tsc1 KO mice and controls at postnatal day (P) 9 are costained with anti-acetylated α-tubulin antibodies (α-Ac-Tub, green) and DBA (red), markers for cilia and the distal tubule/collecting duct, respectively. Cilia length in DBA-positive cells is measured for both pre-cystic (normally shaped tubules) and cystic lesions (area > 2000 µm^2^). (**b**) The aggregate quantification of cilia length in P9 and P11 tubular cells. The average length of pre-cystic, as well as cystic lesions, is compared between control and Cd79a-Tsc1 KO tubules. (**c**) Changes in cilia length over time during cyst expansion in Cd79a-KO tubules. The average cilia length was calculated from the total cilia number (n) including both normally shaped pre-cystic and cystic lesions. Cillia in Cd79a-KO tubules became longer in proportion to cyst expansion and reached a maximum length at 4 weeks of age. These data were obtained from at least 3 litters for different age. Data represent mean ± SEM and were statistically assessed by one-way ANOVA. *P < 0.05 and ****P < 0.0001.

We examined primary cilium length as marked by acetylated α-tubulin. In distal tubules of Cd79a-Tsc1 KO at P9, cilia were significantly longer in cells of the pre-cystic portion, and even longer in those lining overt cysts (an average 16% increase compared to controls, Fig. 8a–c). Over the course of cyst development, cilia in the distal segments continued to lengthen and became even longer (37–92%) during later developmental stages (4 to 9 weeks) relative to controls (Fig. 8c). These results indicate that cilia elongation may be associated with both cyst initiation and expansion. Notably, in Cd79a-Tsc1 KO mice at P9, cilia elongation (24%) was also increased compared to controls in the proximal tubules, which were remote from segments of mTOR activation (Supplementary Fig. S11). At later stages of development (4 to 9 weeks), cilia in the proximal segments were still longer by 10–30% compared to controls, though the extent of lengthening was less than in the distal portion. These observations suggest that cilia length may be regulated in part by non-cell-autonomous mechanisms.

### Aberrant PCP in pre-cystic, Cd79a-Tsc1 KO tubules

**Figure 9:**
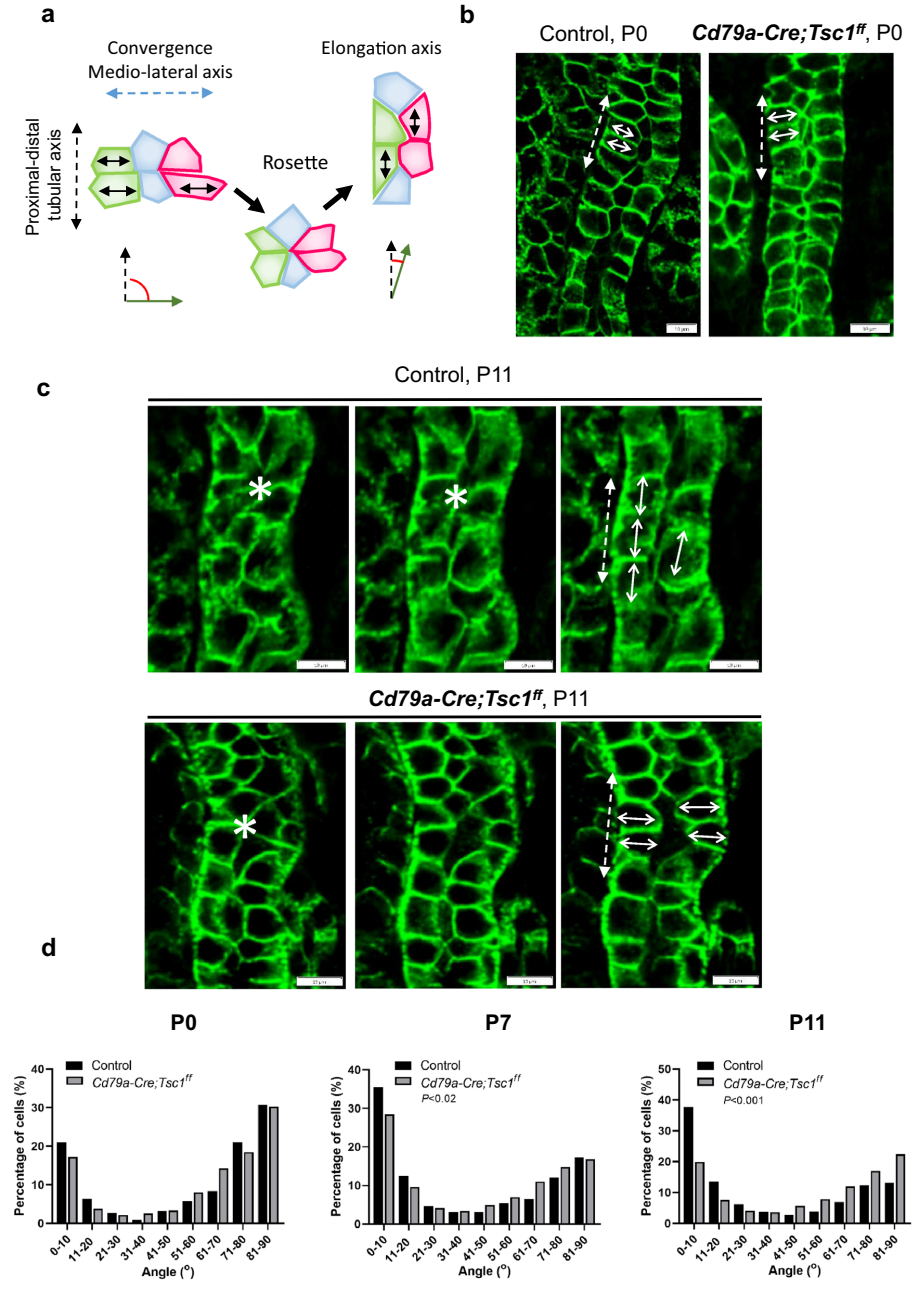
Defective convergent extension (CE) in postnatal, pre-cystic tubules of Cd79a-Tsc1 knockout (KO) kidneys. (**a**) Diagram illustrating the CE process. During tubular formation, the orientation of cell elongation switches from a medio-lateral to a proximal–distal direction through the formation and resolution of an intercalating rosette state. Angles (red) are measured between the cell elongation axis (green arrow) and the tubule elongation vectors (broken black arrow). (**b**) Orientation of cell elongation axis at P0. Section of tubules with double-positive staining for E-cadherin (green) and DAB (not shown) are viewed from the apical side. In both control and Cd79a-Tsc1 KO tubules, the majority of epithelial cells were elongated and their long axes aligned (double-headed arrows) perpendicular to the tubular axis (dot double-headed arrows). (**c**) Orientation of cell elongation axis at P11. Three consecutive Z-stack images from identical tubule sections were evaluated. In controls, long axis of tubular cells was aligned to tubular axis (double-headed arrows) through rosette formation (asterisks). By contrast, in Cd79a-Tsc1 KO tubules, an increasing number of cells elongated mediolaterally to tubular axis (double-headed arrows). (**d**) Histogram showing postnatal changes (P0 to P11) in cell elongation axis. At P0, tubular cells of both control (n = 411) and Cd79a-Tsc1 KO mouse (n = 238) kidneys showed a bimodal distribution of cell elongation axis. About 68% (control) and 73% (Cd79a-Tsc1 KO) of cell elongation axes were perpendicular to tubular axis (within angles 45° and 90°). Over time from P7 to P11, control tubular cells shifted their long axis toward being parallel to tubular axis: 57% (P7, n = 1026) and 61% (P11, n = 796) of cells’ long axes were within an angle between 0° and 45°, thus favoring tubule elongation. By contrast, long axis of Cd79a-Tsc1 KO tubule cells was distributed more randomly. The distribution was significantly different from that of controls (at P7, *P* < 0.02, n = 851; at P11, *P* < 0.001, n = 2076). Data were obtained from a minimum 6 kidneys from at least 3 litters. Statistical differences were determined by the Kolmogorov–Smirnov and Mann–Whitney U tests.

We evaluated the cell elongation orientation of pre-cystic tubules. At P0, cells of both control and Cd79a-Tsc1 KO tubules showed a bimodal distribution of the cell elongation axis. The majority of cell elongation axes, 68% in controls and 73% in Cd79a-Tsc1 KO mice, lay perpendicular to the tubular axis (within angles 45° and 90°) (Fig. 9a,b,d). From P7 to P11, control tubular cells shifted their long axis toward a parallel position relative to the tubular axis: 57% and 61% of the cell long axis lay within an angle of 0° to 45°, thus favouring tubule elongation (Fig. 9c–d). By contrast, the long axis of Cd79a-Tsc1 KO tubule cells was distributed more randomly, which significantly differed from that of controls (Fig. 9c–d). These results indicate that cell intercalation is disrupted in the pre-cystic tubular lining where cells undergo postnatal tubule maturation.

**Figure 10:**
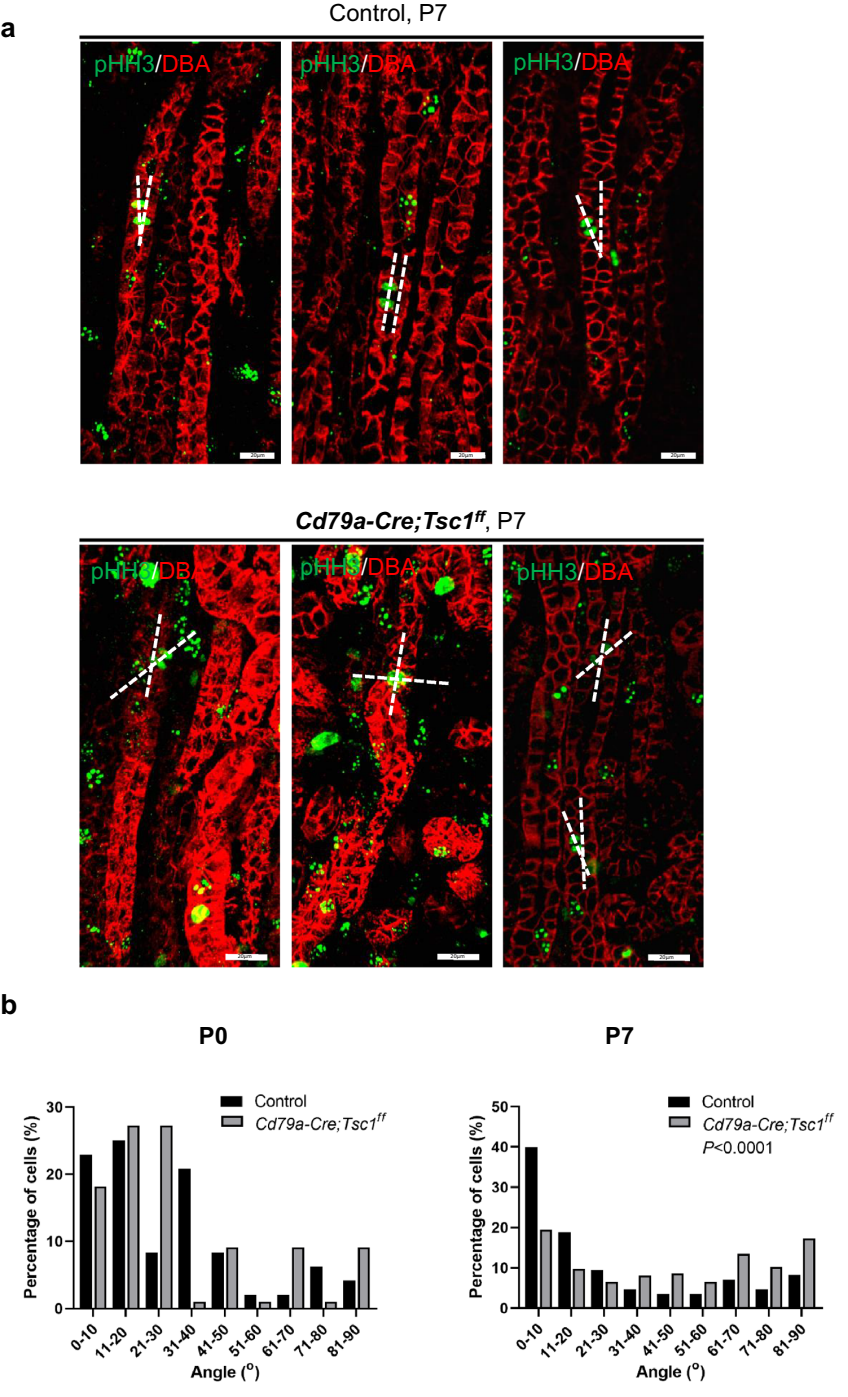
Mitotic orientation in pre-cystic tubules of Cd79a-Tsc1 knockout (KO) kidneys. (**a**) 3D reconstruction of the mitotic orientation of dividing epithelial cells in pre-cystic, normal-looking tubules. Sections of control and Cd79a-Tsc1 KO kidneys were stained with phospho-histone H3 (pHH3, green) and DBA (red), markers for condensing chromosomes in dividing cells and the distal tubule/collecting duct, respectively. Representative images from 3 independent tubules are shown. Mitotic angles and the difference in orientation between mitotic chromosomes and the tubular axis were measured. (**b**) Time-course changes in the distribution of mitotic angles in the dividing tubular cells of the kidney. At P0, Cd79a-Tsc1 KO tubule cells oriented their mitotic spindles along the tubule axis in a manner indistinguishable from that of controls (n = 11 vs n = 48 control, ns). At P7, the mitotic spindle of dividing control tubules was oriented more parallel to the tubular axis: 70% of angles were smaller than 34°, with an average of 11°, are were no longer random. By contrast, the distribution of the mitotic orientation in CD79a-KO tubule cells was significantly distorted, with a more random distribution compared with controls (*P* < 0.001, n = 185 vs control n = 85). These data were obtained from a minimum of 6 kidneys from at least 3 litters. Statistical differences were determined by the Kolmogorov–Smirnov and Mann–Whitney U tests.

We next analyzed the mitotic orientation in pre-cystic tubules. At P0, dividing cells in Cd79a-Tsc1 KO tubules oriented their mitotic directions in a manner indistinguishable from that of controls (Fig. 10a,b). At P7, control tubule cells drastically changed their mitotic axis to be parallel to the tubular axis (Fig. 10b). By contrast, mitotic alignments in CD79a-Tsc1 KO tubule cells were significantly distorted from the normal bias and shifted toward a more random orientation compared with control tubules. Our data indicate that mitotic orientation is perturbed in pre-cystic Cd79a-Tsc1 KO tubule cells during the tubular elongation phase at P7.

## Discussion

The timing of cystogenic triggers is a critical determinant of PKD patterns and severity. Prior studies revealed that tubules undergoing postnatal maturation, specifically between P0 to P13, are more sensitive to the functional loss of regulators of tubule morphogenesis. For example, PKD1 inactivation in newborn mice leads to rapid cyst development, whereas ablating it after P13 leads to slowly progressive adult-onset PKD^20,21^. Cd79a-Tsc1 KO mouse kidney tubules develop normally with no visible cysts at birth, suggesting that aberrant mTOR activation does not affect embryonic tubule development. Moreover, Cd79a-Tsc1 KO tubules proliferate at physiological levels comparable to controls during the critical time window (P0-P14) for cystogenesis. Instead, PCP is distorted in normal-looking tubules at P7-11 before overt cyst appearance. Our results indicate that defective PCP in postnatal stages may be associated with cyst formation in mTOR-activated tubules.

One of our noteworthy findings is that CE was disorganized in postnatal, normally shaped (pre-cystic) tubules. At birth (P0), the long axes of cells in Cd79a-Tsc1 KO tubules showed bimodal distributions indistinguishable from that of age-matched controls, which is consistent with a prior study^30^. Such observations suggest that mTOR activation during embryonic stages neither directly affected PCP signaling nor perturbed tubular narrowing. We further demonstrated that from P7 to P11, control tubules dramatically shifted their cell’s long axis toward the tubular axis, favoring postnatal tubule elongation. In sharp contrast, pre-cystic Cd79a-Tsc1 KO tubule cells from P7 to P11, in which the proliferation rate was comparable to controls, misoriented their cell elongation and mitotic axes and exhibited a more random phenotype. That we saw defective CE in postnatal stages conflicts with a prior observation in a model where the PCP core gene *Vangl2* was inactivated, as in that study there was aberrant CE in embryonic tubules that was restored postnatally^37^. Our results suggest that mTOR regulation is crucial for postnatal maintenance of the CE process. Further study is necessary to clarify the role of PCP signaling in the CE movement of Cd79a-Tsc1 KO tubules.

The role of cilia in PCP regulation is still under debate. In several PKD models, the time of the first cysts emerging (approximately E15.5) coincides with tubule elongation and the beginning of urine excretion, suggesting a link between PCP and cilia function^23,26^. By contrast, in mouse models where the cilia proteins Pkd1/2 or Pkhd1 deleted, defective PCP was only associated with modest tubular dilation, suggesting that there is neither a necessary nor sufficient role of PCP in cystogenesis^30,31,38^. In contrast, in Cd79a-Tsc1 KO mice, pre-cystic tubule cells around P11 showed both distorted CE and OCD and cilia elongation, all of which preceded overt cyst appearance. Consistent with our observation, inactivation of the intraflagellar transport genes *Kif3a* and *Ift20* led to the loss of cilia, aberrant OCD, and cyst formation^39–41^. Disruption of the PCP-related protocadherin *Fat4*, which localizes in the primary cilia, leads to cyst formation, suggesting that cilia may be necessary for OCD to prevent tubular dilation^41^. Our findings, together with those of others, indicate a possible link of PCP to cilia-dependent signaling and cystogenesis.

Defective cilia mouse models show that cystogenesis is often associated with cilia length defects (Supplementary Table S2). In Cd79a-Tsc1 KO kidneys, cilia are elongated in distal tubule cells at P9 before cysts overtly arise, suggesting that cilia length defects may contribute to cyst initiation. Cilia elongation is reported in several cellular and mouse models of Tsc1 depletion^11,42^. Moreover, heterozygous loss of Raptor reversed cilia elongation in our Cd79a-Tsc1 KO tubules at 4 and 6 weeks of age, suggesting that cilia length is regulated by mTORC1 signaling (Fig. 6d). Cilia elongation is observed in tubules of human ADPKD and in PKD1 depletion models, and it coincides with disease progression^43^. These observations indicate that the cilia length defect is a universal feature of cystic disease. Interestingly, we found that cilia were longer even in normally shaped proximal tubules lacking mTOR activation. The broader distribution of length defects beyond programmed mTOR activation suggests that cilia elongation may not simply depend on mTOR signaling^42,44^. Thus, we must consider several mTOR-independent mechanisms for the regulation of cilium morphology. Humoral or signaling factors from neighboring cells and/or the extracellular matrix may affect cilium morphology^24,45,46^. In our model, cysts formed predominantly in the distal portion, indicating that the flow sensor–enriched, upstream proximal portion is prone to secondary obstruction^1,23^. An alternative attractive hypothesis is that Tsc1 signals could be linked to the cilium “flow sensor” polycystin complex, and this should be considered in future studies^10,11,47^.

Our Cd79a-Tsc1 KO mice, in which the primary effect is mTOR activation, show a unique spatiotemporal pattern of cell proliferation while sharing common histopathologic features with other PKD models where PKD1/2 is inactivated and intraflagellar transport is ablated (Supplementary Tables S3 and S4). Regarding the temporal profile of enhanced cell proliferation, Cd79a-Tsc1 KO tubules show persistently increased cell proliferation even after P14, the time point at which programmed tubule maturation is complete^48,49^. Physiologically, postnatal proliferation, which is coordinated with morphogenic processes (CE and OCD) for tubule maturation, abruptly drops at P14^29,49,50^. Aberrant proliferation could trigger cystogenic events, such as distortion of cell–cell or cell–matrix interactions or tubule obstruction due to excessive exfoliation. Enhanced proliferation during the early postnatal period and/or after a physiological drop are also reported for other mouse models^18,51^. In accordance with previous data on spatial preference in cystogenesis, our model shows a PKD phenotype, preferentially arising from distal nephrons. Cd79a-Tsc1 KO kidneys manifest their first cysts around P7, mainly originating from the outer cortex and gradually expanding into the medulla. The distal portion predominance in our mice likely reflects two factors. One is that mTOR activation by Cd79a-Cre in the distal portion is limited from E16 onward. The other is that distal tubule cells have a high cyst formation susceptibility during development because they require the full potential of morphogenic regulators to coordinate proliferation and tubulogenesis^23,45,46^. In line with this hypothesis, the distal portion is the most frequent site of cystogenesis in human ADPKD as well as in other mouse models (Supplementary Tables S3 and S4). Taken together, our model (aberrant proliferation) shares some core features of PKD with the primary cilium disorders. Aberrant mTOR and cilium signaling may converge on a common mechanism triggering cystogenesis: the inability to coordinate proliferation with PCP during the tubule narrowing process.

Our Cd79a-Tsc1 KO mice exhibited the two key features of mTOR activation. One is that mTOR was aberrantly activated in a certain portion of cyst-lining cells. This mosaic feature of cyst-lining cells is in agreement with previous rodent models of Tsc1 and PKD1/2 inactivation^12^. This may depend on the efficiency of Cre recombination. The other key aspect is that mTOR is aberrantly activated even in non-Cre recombinant cells, independent of Tsc1 ablation, pointing to the role of non-autonomous mechanisms in cystogenesis. Mechanical or chemical cues, auto- or paracrine factors, or an altered extracellular matrix might contribute to cystogenesis^36,52^.

Aberrant temporal profiles of mTOR activation lead to various degrees of cysts. For example, embryonic Tsc1 KO mice with Nse-Cre had earlier cyst formation in the proximal nephrons starting at P7^12^. In contrast, postnatal Tsc1 KO mice with Aqp2-Cre exhibited later cyst progression after 4 to 8 weeks of age (Supplementary Table S5). Notably, our model exhibited sustained mTOR activation and tumorous lesions appear after 4 weeks. Moreover, proliferation accompanied by apoptosis is a hallmark of PKD pathogenesis^36^. Physiologically, apoptosis is tightly regulated during nephrogenesis and declines postnatally^36^. Apoptosis promotes cyst cavitation, triggers proliferation, and removes superfluous damaging cells to prevent tumorigenesis. In our Cd79a-Tsc1 KO mice, apoptosis was rarely seen in postnatal tubules until the age of 4 weeks, but was more frequently detected at later stages. Our results suggest that apoptosis itself may not drive cystogenesis and may play a preventive role in tumorigenesis by removing excessive cells.

We have to take into account the possibility that the B cell alteration could influence the cystogenesis in our Cd79a-Tsc1 KO mice, given prior observations that a B-cell specific depletion of Tsc1 activate the mTOR signaling, and that the immune factors generally contribute to the cystogenesis^35,53^. In our Cd79a-Tsc1 KO mice, there are a considerable reduction in the B-cell subpopulation consisting of immature B cells in bone marrow, as well as, FO-B cells and MZ-B cells in the spleen. However, histologic inspection with Cd79a-Tsc1 kidneys also showed no infiltration of mononuclear cells in the tubular interstitium of both embryonic and postnatal cystic tubules. Previous studies on human and rodent PKD models showed that inflammatory response may be seen in the very early stage. Moreover, local mononuclear cell infiltration as well as recruitment of more circulating monocytes and lymphocytes by the chemoattractant often promote cyst growth in later stages^54,55^. Among the mononuclear cells, most studies have focused on the relevance of resident macrophages in promoting cyst progression in animal models through the enhancement of epithelial proliferation and phagocytosis of apoptotic epithelial cells^56^. T cells, a CD4^+^ and CD8^+^ subset, also increasingly accumulate around the cystic lesions even at earlier stages of cystogenesis and are correlative with disease severity onward^53^. In contrast, the role of B cell-related immune dysregulation in cystogenesis still remains unknown. Taken together, our observations indicate that alterations in the development and maturation of the B cell subpopulation in Cd79a-Tsc1 KO mice do not significantly contribute to the initial renal tubular cystogenesis and expansion. Further study is necessary to examine the detailed analysis of B cell behaviors in the Cd79a-Tsc1 KO kidney and to trace the changes until the later stage of cyst formation.

In conclusion, our study indicates that dysregulation of mTOR signals in the dynamic developmental context contributes to initial cystogenesis. Defining the mechanisms orchestrating PCP, cilia signaling, and cell proliferation are essential steps for a better understanding of PKD and the development of earlier intervention strategies that can reduce the burden of cysts in adults.

## Methods

### Animals

The *Tsc1ff.* line was purchased from The Jackson Laboratory (005680)^32^. The floxed *Raptor*^*ff*^, *ROSA26-tandem-dimer RFP*^*ff*^ reporter and the Cd79a (mb1)-Cre lines were generously gifted from investigators^57,58^. All animals were housed, maintained, and studied according to approved protocols from the Institutional Animal Care and Use Committee at the Kansai Medical University (Assurance Number 20-120). All mouse lines were fully inbred into the C57BL/6J strain. Animals were sacrificed by inhalation of isoflurane (Pfizer). Kidneys were fixed in 4% paraformaldehyde and embedded into paraffin. In postnatal tubules (from P0 to P14), a cyst was defined as > 2000 µm^2^, and a dilated tubule was discernible as a luminal area of > 500 µm^2^^59^. After early adulthood (~ P22), a luminal area of 2000 µm^2^ was regarded as a cyst^43^. Cystic index was measured as the ratio of cystic area to the total section area.

### Immunostaining, proliferation, and apoptosis

See Supplementary Table S1.

### Immunoblot analysis

Isolated kidneys were Dounce-homogenized in lysis buffer including 150 mM NaCl, 20 mM Na_2_HPO_4_/NaH_2_PO_4_, 10% glycerol, 1% Triton X-100, phosphatase inhibitors (0.5 M NaF, 0.5 M β-glycerophosphate disodium, 100 mM Na_3_VO_4_), and protease inhibitors. After centrifugation at 12 000 rpm for 20 min, the supernatant was electrophoresed on a 4–12% Bis–Tris Plus gel (Invitrogen) and then transferred to PVDF by Trans-Blot-Turbo.

### Orientation of CE and OCD

Paraffin sections (15-µm thick) were stained with DBA and E-cadherin or anti-phospho-histone H3 antibody, and normal-looking tubules were evaluated with positive DBA staining. Z-stack image series were captured at every 1 µm of thickness by an FV3000 confocal microscope. For CE evaluation, cells with E-cadherin staining that outlined the entire cell and in which the length-to-width ratio was > 1.2 were judged as “elongated.” For all elongated cells, the angle between the longest axis of the individual elongated cells and the longitudinal tubular axis was calculated by ImageJ^28^. The percentage of total cells within each 10° different was calculated. For OCD evaluation, the mitotic angle was measured by calculating the angle between the separating chromosome and the longitudinal tubular axis and making a three-dimensional reconstruction by Olympus cellSens software.

### Reverse transcriptase PCR quantification of Cd79a transcript

Total RNA was isolated from whole mouse embryos with a Qiagen RNA kit and was reverse transcribed using a cDNA synthesis kit (Thermo Fisher). cDNA (25 ng) was amplified using the primer set for Cd79a (sense: c.61–309 and antisense: c.848–1270, in NM_007655.3) under different PCR cycling conditions (25, 30, 35 cycles). The resulting PCR products (600 bp) were electrophoresed on a 1% agarose gel.

### FACS analysis

A single-cell suspension was prepared using the Tumor Dissociation Kit according to the manufacturer's protocol (Miltenyi Biotec, Bergisch Gladbach, Germany). Cells were further treated by Ammonium-Chloride-Potassium (ACK) Lysing Buffer, and lysates were analyzed by the FACSCanto II (BD Bioscience, USA).

Antibodies used in FACS analysis were as follows: B220 (RA3-6B2), TCRβ (H57-597), CD21/CD35 (7E9), and CD23 (B3B4) from Biolegend; IgM (R6-60.2) from BD Biosciences. Single-cell suspensions from the bone marrow and the spleen were blocked with 10 µg/ml of anti-mouse CD16/CD32 (2.4G2; BD Biosciences) and then stained with fluorochrome-conjugated antibodies along with 7-aminoactinomycin D (7-AAD; Sigma) to exclude dead cells. Data were acquired on a FACSCanto II (BD Biosciences) and analyzed with BD FACSDiva software (BD Biosciences).

### ELISA

Serum was collected from control (n = 3) and Cd79a-Tsc1-KO (n = 3) mice at 2 months of age. Serum IgM and total IgG, and subtypes (IgG1, IgG2b, IgG2c, and IgG3) concentrations were determined by ELISA using SBA Clonotyping System-C57BL/6-HRP (Southern Biotech).

### Ethics declarations

All animals were housed, maintained, and studied according to approved protocols from the Institutional Animal Care and Use Committee at the Kansai Medical University (Assurance Number 20-120). All methods were performed in accordance with the relevant guidelines and regulations. The study is reported in accordance with ARRIVE guidelines.

## Supplementary Information

Supplementary Information.

## Data Availability

Datasets used and/or analyzed during the current study available from the corresponding author on reasonable request.

## References

[1] Bergmann C, Guay-Woodford LM, Harris PC, Horie S, Peters DJM, Torres VE (2018). Polycystic kidney disease. Nat. Rev. Dis. Primers..

[2] Grantham JJ (2008). Clinical practice. Autosomal dominant polycystic kidney disease. N. Engl. J. Med..

[3] Pei Y (2011). Practical genetics for autosomal dominant polycystic kidney disease. Nephron. Clin. Pract..

[4] Churchill DN, Bear JC, Morgan J, Payne RH, McManamon PJ, Gault MH (1984). Prognosis of adult onset polycystic kidney disease re-evaluated. Kidney Int..

[5] Peters DJ, Breuning MH (2001). Autosomal dominant polycystic kidney disease: Modification of disease progression. Lancet.

[6] Fick GM (1993). Characteristics of very early onset autosomal dominant polycystic kidney disease. J. Am. Soc. Nephrol..

[7] Pretorius DH, Lee ME, Manco-Johnson ML, Weingast GR, Sedman AB, Gabow PA (1987). Diagnosis of autosomal dominant polycystic kidney disease in utero and in the young infant. J. Ultrasound. Med..

[8] Armour EA, Carson RP, Ess KC (2012). Cystogenesis and elongated primary cilia in Tsc1-deficient distal convoluted tubules. Am. J. Physiol. Renal. Physiol..

[9] Saxton RA, Sabatini DM (2017). mTOR Signaling in growth, metabolism, and disease. Cell.

[10] Shillingford JM (2006). The mTOR pathway is regulated by polycystin-1, and its inhibition reverses renal cystogenesis in polycystic kidney disease. Proc. Natl. Acad. Sci. U. S. A..

[11] Hartman TR (2009). The tuberous sclerosis proteins regulate formation of the primary cilium via a rapamycin-insensitive and polycystin 1-independent pathway. Hum. Mol. Genet..

[12] Zhou J, Brugarolas J, Parada LF (2009). Loss of Tsc1, but not Pten, in renal tubular cells causes polycystic kidney disease by activating mTORC1. Hum. Mol. Genet..

[13] Kumar P (2022). Tsc2 mutation induces renal tubular cell nonautonomous disease. Genes Dis..

[14] Kim HJ, Edelstein CL (2012). Mammalian target of rapamycin inhibition in polycystic kidney disease: From bench to bedside. Kidney Res. Clin. Pract..

[15] Holditch SJ (2019). A study of sirolimus and mTOR kinase inhibitor in a hypomorphic Pkd1 mouse model of autosomal dominant polycystic kidney disease. Am. J. Physiol. Renal. Physiol..

[16] Watnick T, Germino GG (2010). mTOR inhibitors in polycystic kidney disease. N. Engl. J. Med..

[17] Wu G (1998). Somatic inactivation of Pkd2 results in polycystic kidney disease. Cell.

[18] Happe H (2013). Cyst expansion and regression in a mouse model of polycystic kidney disease. Kidney Int..

[19] Smith LA (2006). Development of polycystic kidney disease in juvenile cystic kidney mice: Insights into pathogenesis, ciliary abnormalities, and common features with human disease. J. Am. Soc. Nephrol..

[20] Leonhard WN, Happe H, Peters DJ (2016). Variable cyst development in autosomal dominant polycystic kidney disease: The biologic context. J. Am. Soc. Nephrol..

[21] Piontek K, Menezes LF, Garcia-Gonzalez MA, Huso DL, Germino GG (2007). A critical developmental switch defines the kinetics of kidney cyst formation after loss of Pkd1. Nat. Med..

[22] Leonhard WN (2015). Scattered deletion of PKD1 in kidneys causes a cystic snowball effect and recapitulates polycystic kidney disease. J. Am. Soc. Nephrol..

[23] Starremans PG (2008). A mouse model for polycystic kidney disease through a somatic in-frame deletion in the 5' end of Pkd1. Kidney Int..

[24] Wang S, Dong Z (2013). Primary cilia and kidney injury: Current research status and future perspectives. Am. J. Physiol. Renal. Physiol..

[25] Happe H, de Heer E, Peters DJ (2011). Polycystic kidney disease: The complexity of planar cell polarity and signaling during tissue regeneration and cyst formation. Biochim. Biophys. Acta.

[26] Torban E, Sokol SY (2021). Planar cell polarity pathway in kidney development, function and disease. Nat. Rev. Nephrol..

[27] McNeill H (2009). Planar cell polarity and the kidney. J. Am. Soc. Nephrol..

[28] Karner CM, Chirumamilla R, Aoki S, Igarashi P, Wallingford JB, Carroll TJ (2009). Wnt9b signaling regulates planar cell polarity and kidney tubule morphogenesis. Nat. Genet..

[29] Fischer E (2006). Defective planar cell polarity in polycystic kidney disease. Nat. Genet..

[30] Kunimoto K, Bayly RD, Vladar EK, Vonderfecht T, Gallagher AR, Axelrod JD (2017). Disruption of core planar cell polarity signaling regulates renal tubule morphogenesis but is not cystogenic. Curr. Biol..

[31] Nishio S (2010). Loss of oriented cell division does not initiate cyst formation. J. Am. Soc. Nephrol..

[32] Hobeika E (2006). Testing gene function early in the B cell lineage in mb1-cre mice. Proc. Natl. Acad. Sci. U. S. A..

[33] Centini R (2018). Loss of Fnip1 alters kidney developmental transcriptional program and synergizes with TSC1 loss to promote mTORC1 activation and renal cyst formation. PLoS ONE.

[34] Benhamron S, Pattanayak SP, Berger M, Tirosh B (2015). mTOR activation promotes plasma cell differentiation and bypasses XBP-1 for immunoglobulin secretion. Mol. Cell Biol..

[35] Ci X (2015). TSC1 promotes B cell maturation but is dispensable for germinal center formation. PLoS ONE.

[36] Ho J (2014). The regulation of apoptosis in kidney development: Implications for nephron number and pattern?. Front. Pediatr..

[37] Derish I (2020). Differential role of planar cell polarity gene Vangl2 in embryonic and adult mammalian kidneys. PLoS ONE.

[38] Papakrivopoulou E, Jafree DJ, Dean CH, Long DA (2021). The biological significance and implications of planar cell polarity for nephrology. Front. Physiol..

[39] Jonassen JA, San Agustin J, Follit JA, Pazour GJ (2008). Deletion of IFT20 in the mouse kidney causes misorientation of the mitotic spindle and cystic kidney disease. J. Cell Biol..

[40] Patel V (2008). Acute kidney injury and aberrant planar cell polarity induce cyst formation in mice lacking renal cilia. Hum. Mol. Genet..

[41] Saburi S (2008). Loss of Fat4 disrupts PCP signaling and oriented cell division and leads to cystic kidney disease. Nat. Genet..

[42] Bonnet CS, Aldred M, von Ruhland C, Harris R, Sandford R, Cheadle JP (2009). Defects in cell polarity underlie TSC and ADPKD-associated cystogenesis. Hum. Mol. Genet..

[43] Shao L (2020). Genetic reduction of cilium length by targeting intraflagellar transport 88 protein impedes kidney and liver cyst formation in mouse models of autosomal polycystic kidney disease. Kidney Int..

[44] Yuan S, Li J, Diener DR, Choma MA, Rosenbaum JL, Sun Z (2012). Target-of-rapamycin complex 1 (Torc1) signaling modulates cilia size and function through protein synthesis regulation. Proc. Natl. Acad. Sci. U. S. A..

[45] Besschetnova TY, Kolpakova-Hart E, Guan Y, Zhou J, Olsen BR, Shah JV (2010). Identification of signaling pathways regulating primary cilium length and flow-mediated adaptation. Curr. Biol..

[46] Seeger-Nukpezah T, Golemis EA (2012). The extracellular matrix and ciliary signaling. Curr. Opin. Cell Biol..

[47] Pema M (2016). mTORC1-mediated inhibition of polycystin-1 expression drives renal cyst formation in tuberous sclerosis complex. Nat. Commun..

[48] Hartman HA, Lai HL, Patterson LT (2007). Cessation of renal morphogenesis in mice. Dev. Biol..

[49] Marquez MG, Cabrera I, Serrano DJ, Sterin-Speziale N (2002). Cell proliferation and morphometric changes in the rat kidney during postnatal development. Anat. Embryol. (Berl.).

[50] Sharma N (2013). Proximal tubule proliferation is insufficient to induce rapid cyst formation after cilia disruption. J. Am. Soc. Nephrol..

[51] Jonassen JA, SanAgustin J, Baker SP, Pazour GJ (2012). Disruption of IFT complex A causes cystic kidneys without mitotic spindle misorientation. J. Am. Soc. Nephrol..

[52] Nowak KL, Edelstein CL (2020). Apoptosis and autophagy in polycystic kidney disease (PKD). Cell. Signal..

[53] Zimmerman KA, Hopp K, Mrug M (2020). Role of chemokines, innate and adaptive immunity. Cell. Signal..

[54] Ta MH, Harris DC, Rangan GK (2013). Role of interstitial inflammation in the pathogenesis of polycystic kidney disease. Nephrology (Carlton).

[55] Karihaloo A (2011). Macrophages promote cyst growth in polycystic kidney disease. J. Am. Soc. Nephrol..

[56] Li Z, Zimmerman KA, Yoder BK (2021). Resident macrophages in cystic kidney disease. Kidney.

[57] Hoshii T (2012). mTORC1 is essential for leukemia propagation but not stem cell self-renewal. J. Clin. Invest..

[58] Luche H, Weber O, Nageswara Rao T, Blum C, Fehling HJ (2007). Faithful activation of an extra-bright red fluorescent protein in “knock-in” Cre-reporter mice ideally suited for lineage tracing studies. Eur. J. Immunol..

[59] Arroyo J (2021). The genetic background significantly impacts the severity of kidney cystic disease in the Pkd1(RC/RC) mouse model of autosomal dominant polycystic kidney disease. Kidney Int..

