# Supplementary material for: Mechanism of cystogenesis by Cd79a-driven, conditional mTOR activation in developing mouse nephrons
Source: Sci Rep. 2023 Jan 10;13:508. doi: 10.1038/s41598-023-27766-2 (PMC9832032; doi:10.1038/s41598-023-27766-2)
Supplement: Supplementary file 1 — Supplementary Information. [file 41598_2023_27766_MOESM1_ESM.pdf]

Table of Content for Supplementary Information

**Supplementary files**	**Descriptive title**
**Supplementary Fig.S1**	FACS analysis of lymphocytes in Cd79a-Tsc1 KO mice.
**Supplementary Fig.S2**	Effect of Tsc1-deficiency on serum immunoglobulin levels in Cd79a-Tsc1 KO mice.
**Supplementary Fig.S3**	Systematic histopathological examinations for whole body organs of Cd79a-Tsc1 KO mice.
**Supplementary Fig.S4**	Histopathological examination for kidneys tissue of Cd79a-Tsc1 KO mice.
**Supplementary Fig.S5**	Segmental origin of cysts in postnatal Cd79a-Tsc1 KO kidneys
**Supplementary Fig.S6**	cDNA microarray analysis of Cd79a (mb1) expression in mouse embryo in public database of GUDMAP.
**Supplementary Fig.S7**	Quantitative Analysis of Cd79a (mb1) mRNA expression in mouse whole embryos.
**Supplementary Fig.S8**	Cd79a (mb1)-Cre driven RFP reporter is expressed in a subpopulation of kidney epithelial cells.
**Supplementary Fig.S9**	Activation of mTOR pathways in Cd79a-Tsc1 KO kidney.
**Supplementary Fig.S10**	P-S6 expression of in tubules of Cd79-Tsc1 KO kidney.
**Supplementary Fig.S11**	Cilia elongation in DBA-negative, proximal tubule cells.
**Supplementary Figure S12**	The original images for cropped gel are presented in Figure 5.
**Supplementary Figure S13**	The original images for cropped gel are presented in Figure 6.

**Supplementary tables**	**Descriptive title**
**Supplementary Table S1**	List of Primary Antibody used in this study.
**Supplementary Table S2**	Relationship between defective PCP and cystogenesis in the cilium-associated mutant models.
**Supplementary Table S3**	Comparison of *Tsc1* versus *PKD1/2* inactivation model of mouse and human.
**Supplementary Table S4**	Comparison of PKD phenotype due to Primary Cilia Defects vs mTOR activation.
**Supplementary Table S5**	Comparison of *Tsc1/2* inactivation model generated by various Cre-promoters.

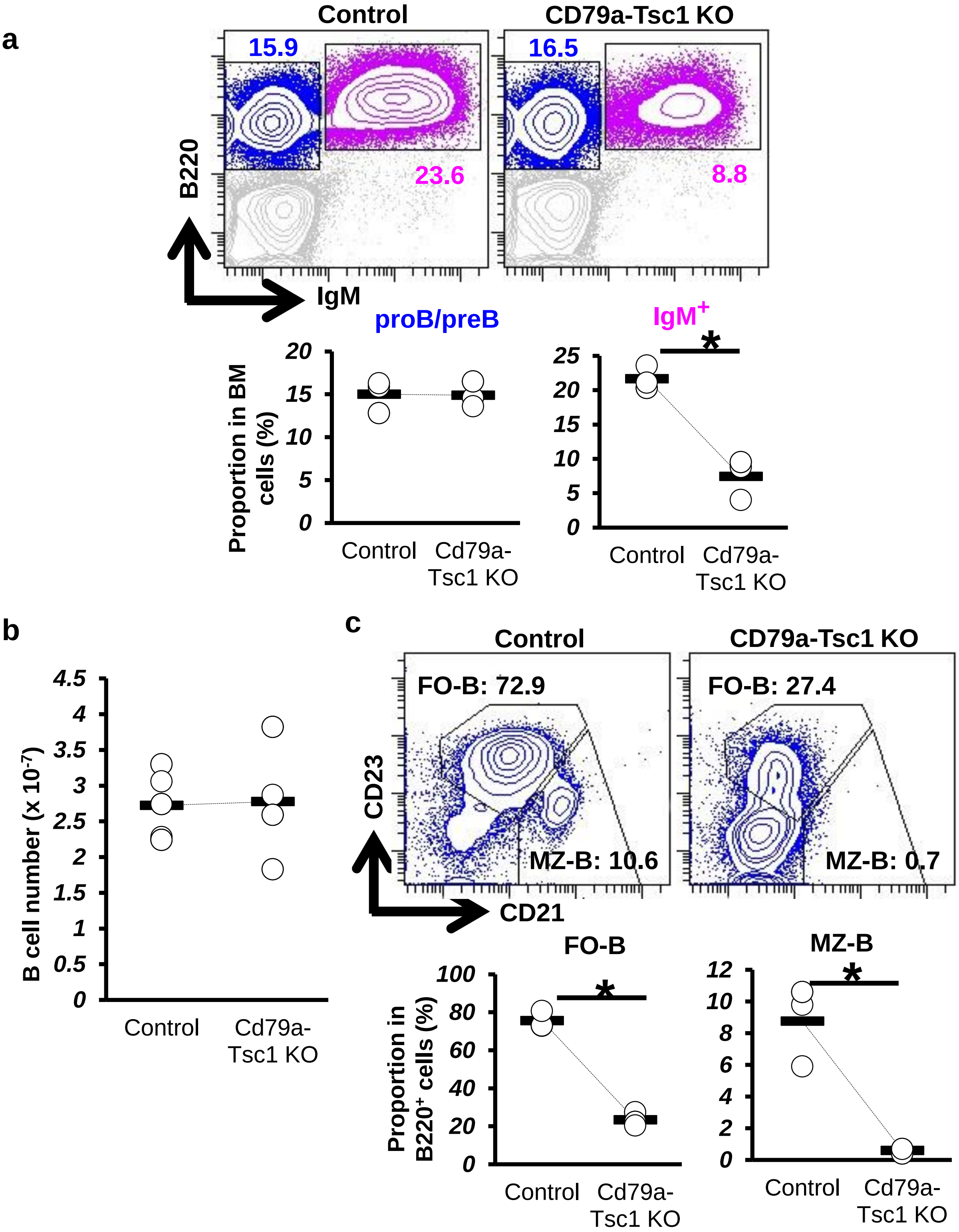

Supplementary Fig S1. FACS analysis of lymphocytes in Cd79a-Tsc1 KO mice.
(**a**) **Characteristics of bone marrow cells by FACS.** A proportion of $B220^{dull}IgM^{-}$ fraction (proB/preB) as well as $B220^{high}IgM^{+}$ fraction (IgM^{+}) in total bone marrow cells are evaluated. Shown are representative FACS profiles (top, number represents percentage) and a group-comparison of the proportions (bottom). **P*< 0.05. (**b**) **Splenic B cell numbers.** The B cell numbers of $B220^{+}TCRb^{-}$ fraction in the spleen are evaluated. (**c**) **Characteristics of splenic B cells by FACS.** A proportion of follicular B cells (FO-B; $CD23^{high}CD21^{dull}$) as well as marginal zone B cells (MZ-B; $CD23^{high}CD21^{high}$) in total $B220^{+}$ cells are evaluated. Shown are representative FACS profiles (top) and a group-comparison of the proportions (bottom). Data are shown by means from 3-5 independent experiments. **P*< 0.05.

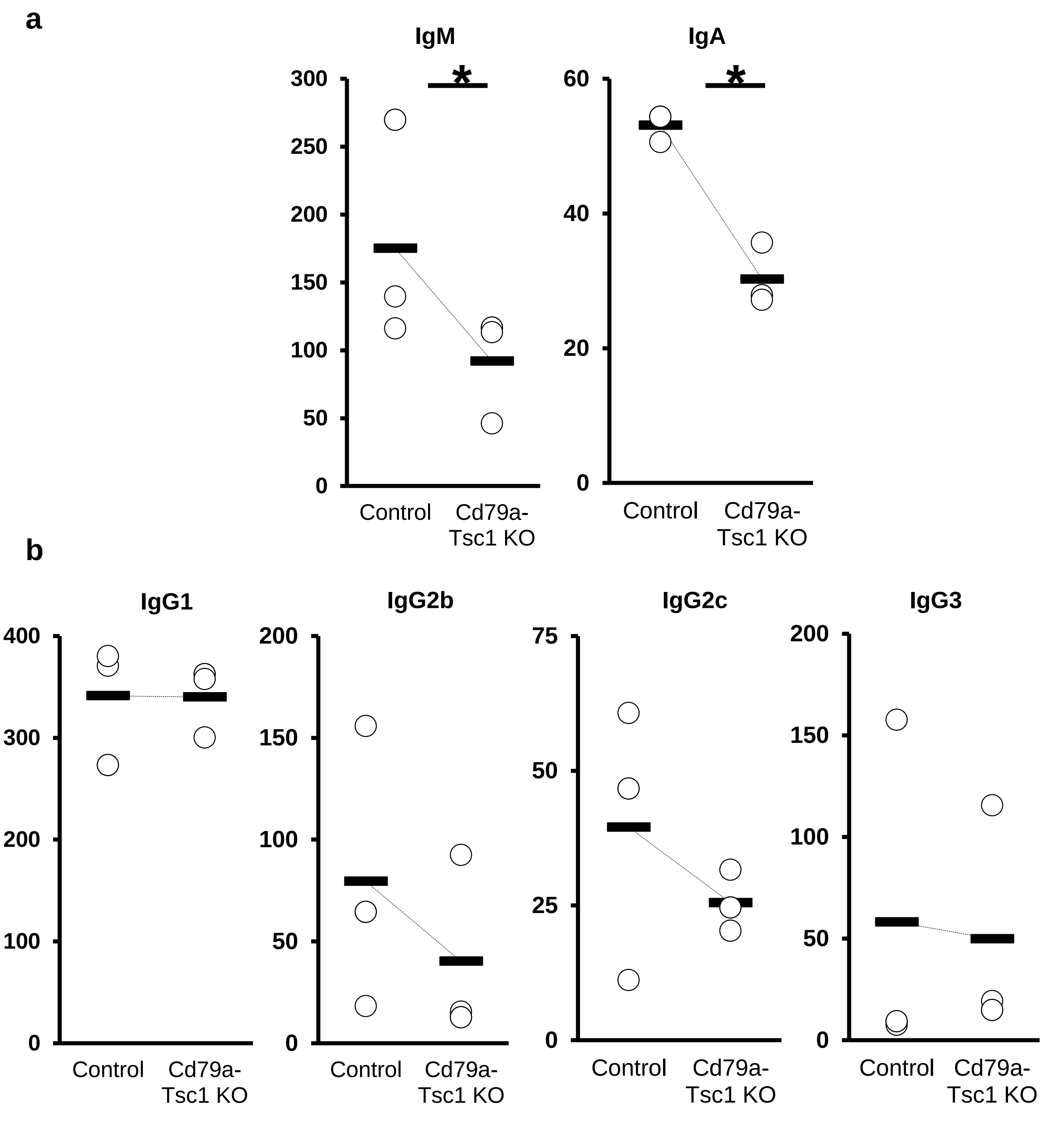

Supplementary Fig S2. Effect of Tsc1-deficiency on serum immunoglobulin levels in Cd79a-Tsc1 KO mice. Serum immunoglobulin levels of IgM, IgA (a) and IgG subtypes (b) in control (n=3) and CD79a-Tsc1-KO (KO, n=3) mice under steady state conditions are evaluated by ELISA. Data are shown by means from three independent experiments. *$P< 0.05$.

a

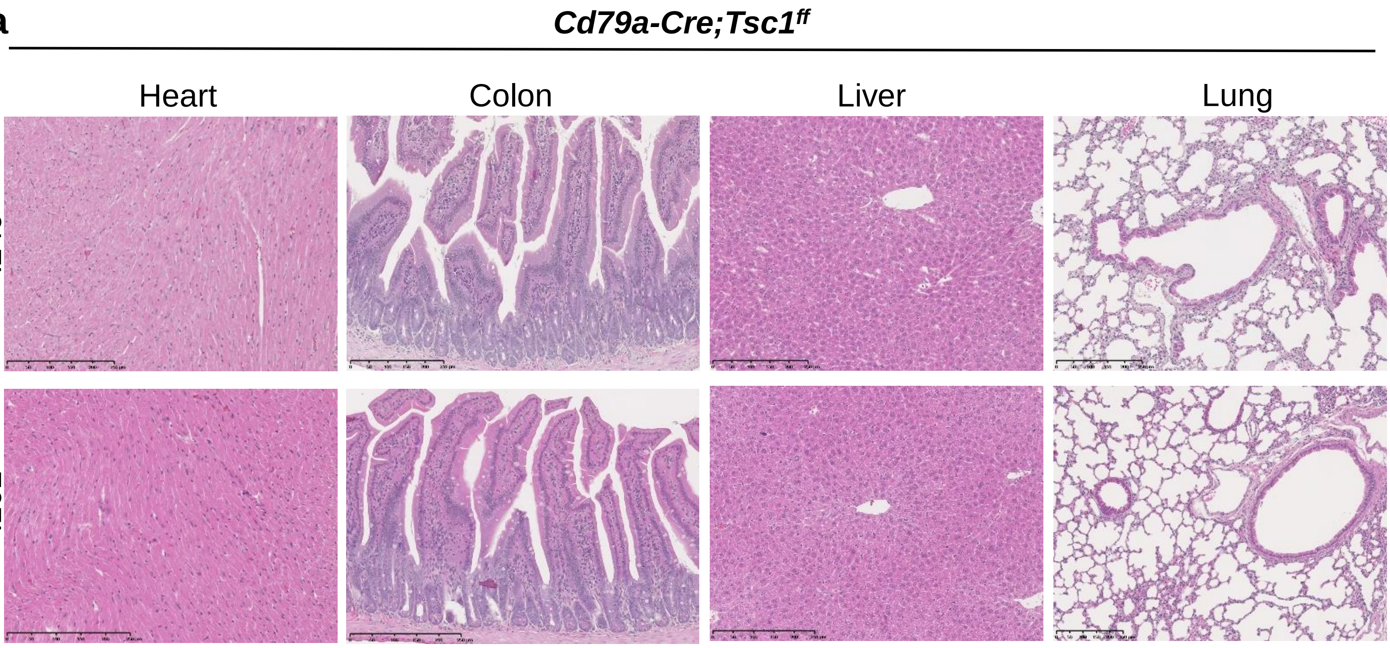

b

Animal ID	i249	f993	f985	i102	g693	g354	g355	g356
Age (Postnatal)	19W		21W	22W	25W			
Gender	F	F	F	F	F	F	F	F
Kidney	PKD	PKD	PKD	PKD	PKD	PKD	PKD	PKD
Liver	–	–	–	–	–	–	–	–
Gall bladder	–	–	–	–	–	–	–	–
Lung	–	–	–	–	–	–	–	–
Heart	–	Inflammation*	–	–	–	–	–	–
Spleen	–	–	–	–	–	–	–	–
Salivary Gland	–	–	–	–	–	–	–	–
Stomach	–	–	–	–	–	–	–	–
S.intestine	–	–	–	–	–	–	–	–
Colon	–	–	–	–	–	–	–	–
Pancreas	–	–	–	–	–	Peritonitis*	–	–
Mesenteric Lymph Node	–	–	–	–	–	–	–	–
Brain	–	–	–	–	–	–	–	–
Testis	–	–	–	–	–	–	–	–
Prostate	–	–	–	–	–	–	–	–
Skin	–	–	–	–	–	–	–	–
Skeletal M	–	–	–	–	–	–	–	–
Eye	–	–	–	–	–	–	–	–

Supplementary Fig S3. Systematic histopathological examinations for whole body organs of Cd79a-Tsc1 KO mice. (**a**) Hematoxylin and eosin stain of paraffin-sections of systemic organs at age from 19 and 22 weeks. Representative histology of heart, large intestine, liver, and lung are shown. There are no infiltration of mononuclear cells. (**b**) Summary histopathological findings of Cd79a-Tsc1 KO mice at age from 19-25 weeks. There are essentially no extrarenal inflammatory phenotypes, with the exception of only occasional inflammatory reactions (asterisks). Scale bars: 250µm. F: Female, PKD: Polycystic kidney. S. intestine: Small intestine. Skeletal M: Skeletal muscle. “-”: indicates the absence of inflammatory regions.

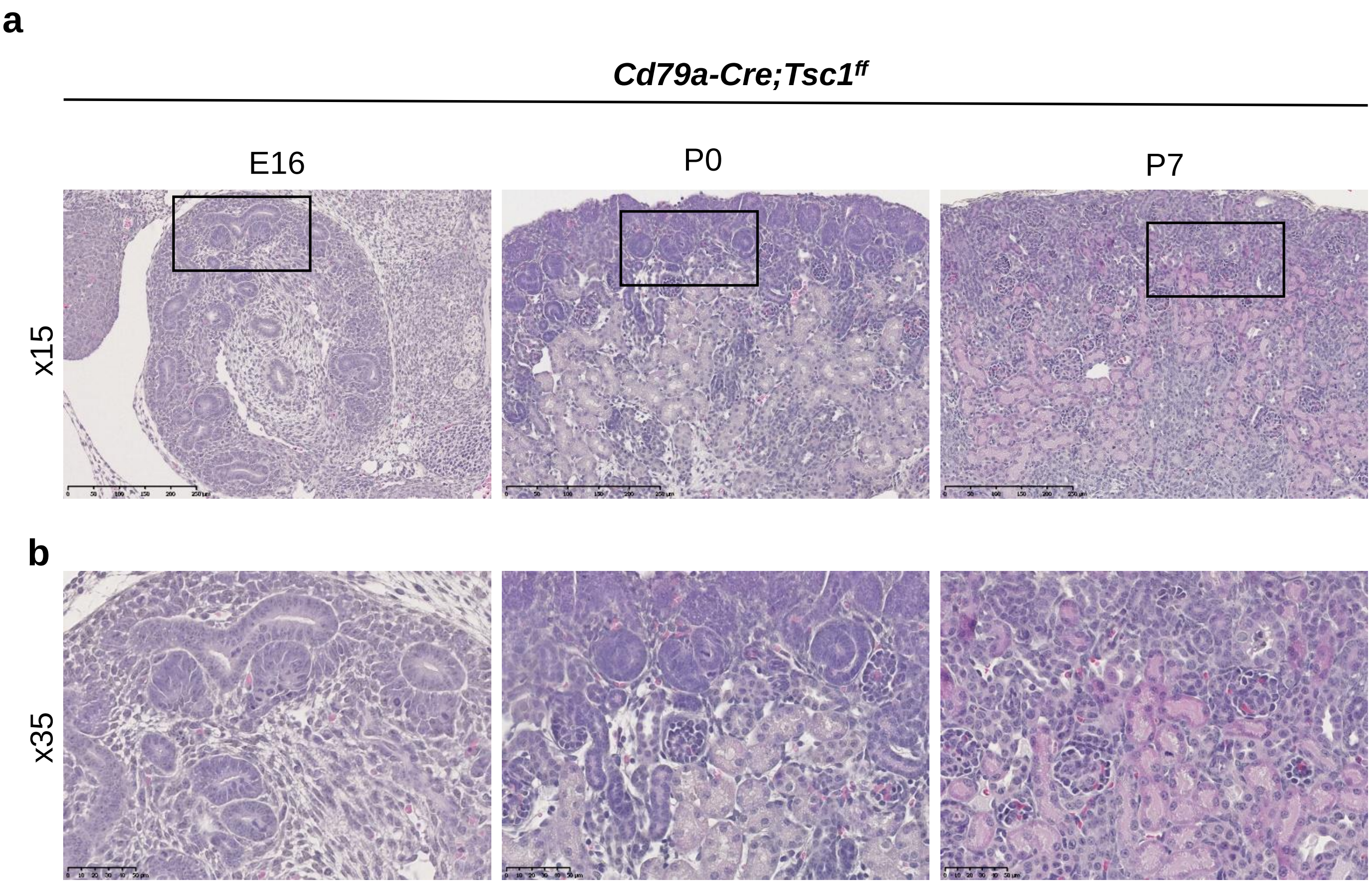

Supplementary Fig S4. Histopathological examination for kidney tissues in Cd79a-Tsc1 KO mice. Hematoxylin and eosin stain of paraffin-sections of the kidneys at age embryonic 16, postnatal day 0, and day 7 with low magnification x15 (**a**) and higher magnification of boxed area x35 (**b**). There is no infiltration of mononuclear cells in the tubular interstitium of the embryonic and postnatal pre-cystic tubules. Scale bars: 50µm (bottom) and 250µm (upper).

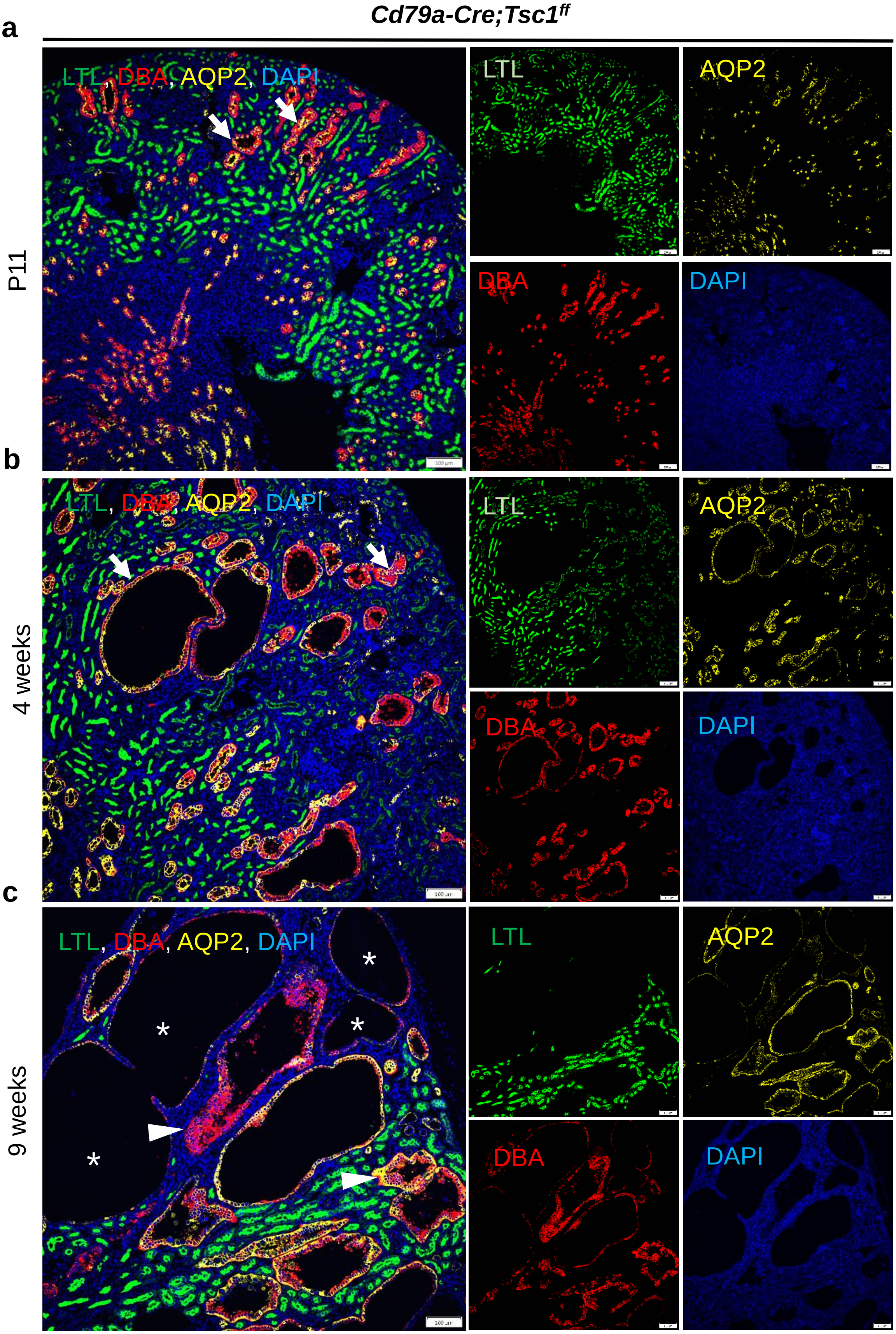

Supplementary Fig S5. Segmental origin of cysts in postnatal Cd79a-Tsc1 KO kidneys. Sections of P11 **(a)**, age of 4 weeks **(b)** and 9 weeks **(c)** of Cd79a-Tsc1 KO kidneys are stained with markers for proximal tubular (LTL, green), distal/collecting tubular (DBA, red), and collecting ducts (AQP2, yellow). Most cysts are double-positive with both DBA and AQP2 (arrows), indicating that cysts mainly originate from the distal and collecting tubules. At age 9 weeks, some cyst-lining cells focally form a multilayer lesion with active proliferation (arrowheads). Dedifferentiation of cyst-lining epithelial cells is evident by the absence of AQP2 and DBA (asterisks). Scale bar: 100µm.

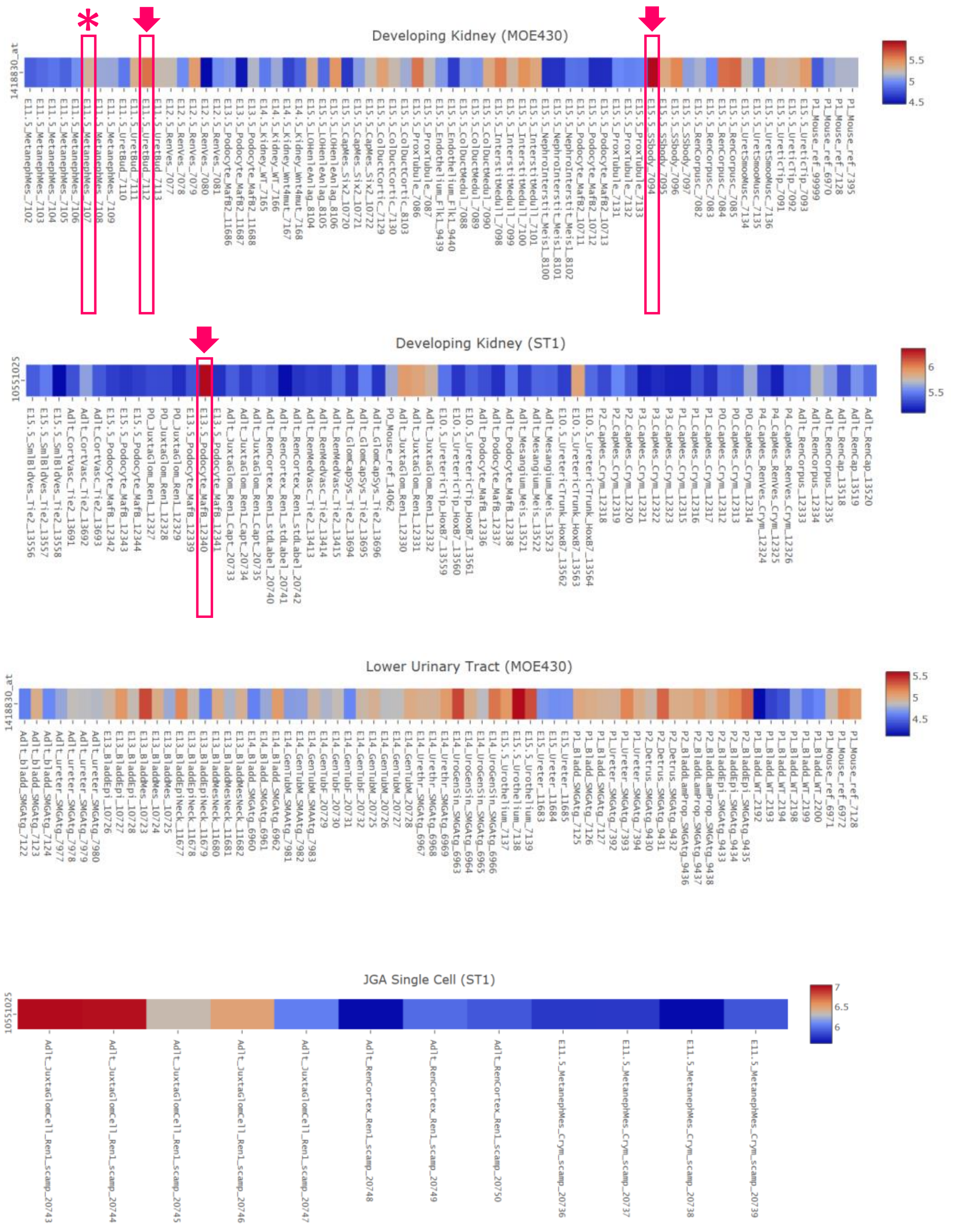

Supplementary Figure S6. cDNA microarray analysis of Cd79a (mb1) expression in mouse embryo in public database of GUDMAP.

Cd79a (mb1) transcripts first appear in developing nephrons at embryonic day (E) 11.5 (asterisk). The highest expression is found between E13.5 and E15.5 in ureteric buds , S-shaped bodies, and podocytes (arrows). Mouse Embryonal Kidney Expression was referred to in the GenitoUrinary Development Molecular Anatomy Project (GUDMAP). https://www.gudmap.org/chaise/record/#2/Common:Gene/RID=Q-47DW

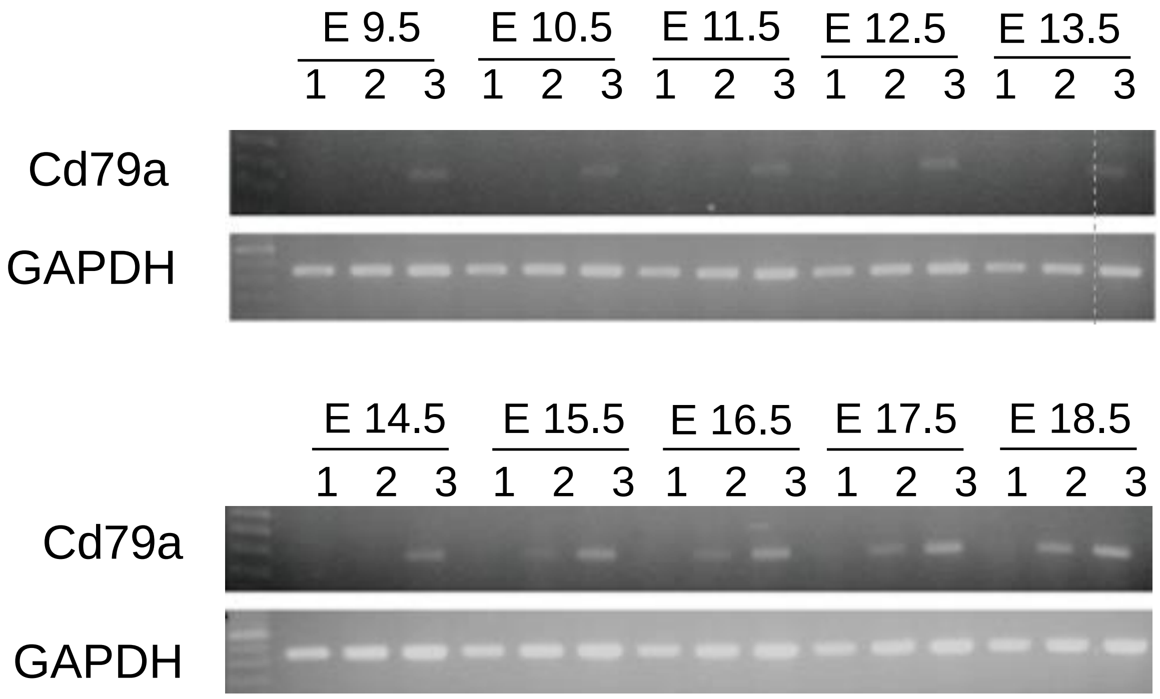

Supplementary Figure S7. Quantitative Analysis of Cd79a (mb1) mRNA expression in mouse whole embryos.

mRNA expression of Cd79a was analyzed by RT-PCR along the developing timeline of mouse embryos: earlier stage from E9.5 to E 13.5 (days post coitum, upper panel) and later stage from E 14.5 to E18.5 (lower panel). Total RNA isolated from whole embryos was reverse-transcribed to cDNA, 25 μg of which was further amplified by Cd79a cDNA primer set under three different cycle conditions (1: 25 cycles, 2: 30 cycles, 3: 35 cycles). Glyceraldehyde 3-phosphate dehydrogenase (GADPH) was used for internal control. Cd79a mRNA is expressed weakly in earlier stages (E 9.5 to E 13.5) and is more abundant in the later stages (E14.5 to E 18.5).

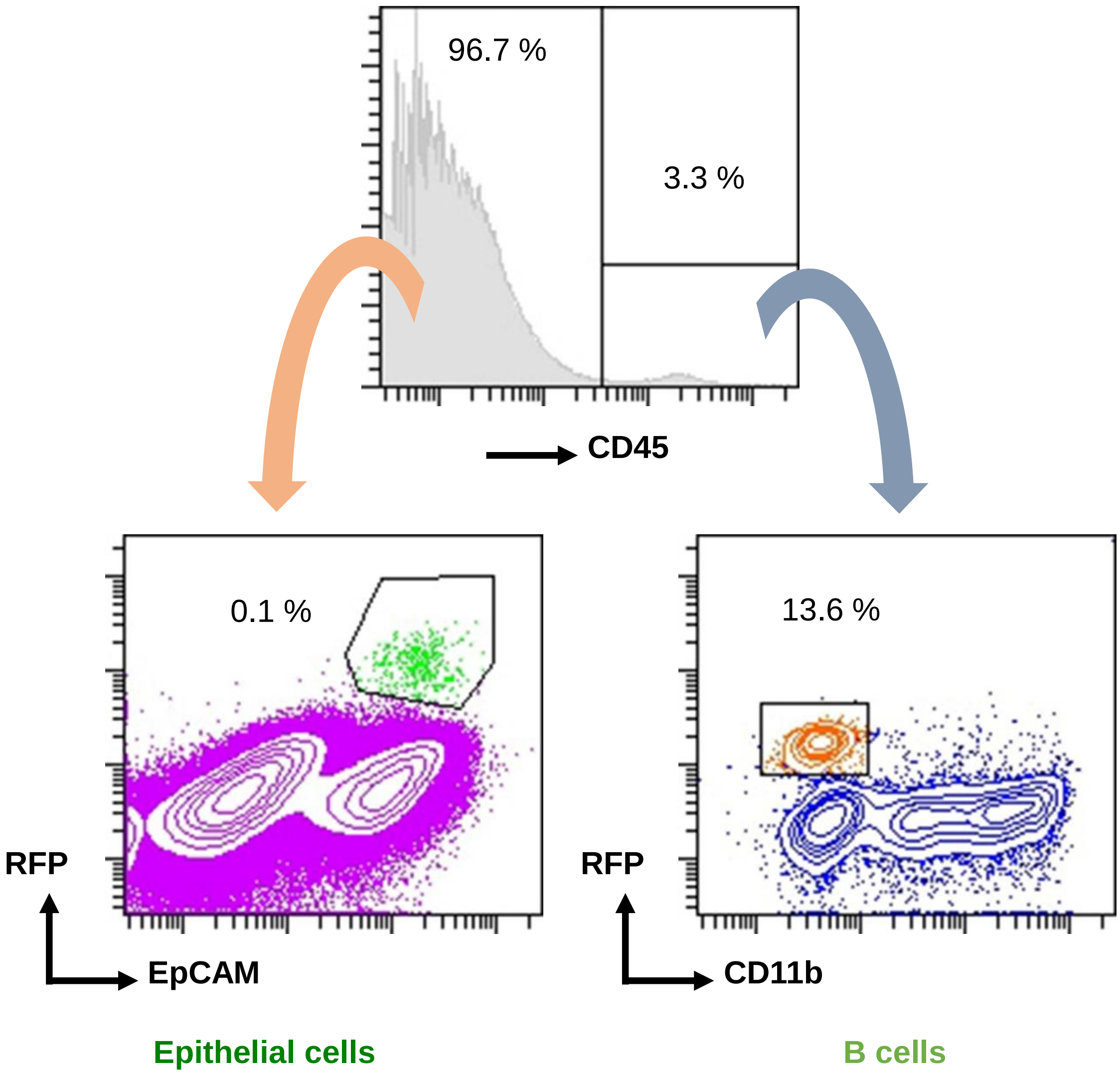

Primary Marker	Fraction (%)	Second sorting markers	Fraction (%)
$CD45^+$	3.3%	$CD11b^+$	RFP^+ 13.6 %<br>RFP^- 86.4 %
$CD45^-$	96.7%	$EpCAM^+$	RFP^+ 0.1 %<br>RFP^- 99.9 %

Supplementary Fig S8. Cd79a (mb1)-Cre driven RFP reporter is expressed in a subpopulation of kidney epithelial cells.

A single-cell suspension was prepared from kidneys from *Cd79a-Cre;Tsc1$^{f/+}$;RFP$^{+/-}$* mice at age of 3 months and was analyzed by FACSCanto II (BD Biosciences, USA). Total cells were first fractionated by marking the CD45, a leukocyte common antigen, which yielded 3.3% positive cells (upper panel). $CD45^+$ and $CD45^-$ cells were further sorted with lineage-specific markers, CD11b (αM integrin, immune cell marker, lower right), and EpCAM (epithelial cell adhesion molecule, an epithelial cell marker, lower left), respectively. RFP-positive cells (RFP^+) account for 0.1% and 13.6% of $CD45^-$/$EpCAM^+$ and $CD45^+$/CD11b subpopulation, respectively.

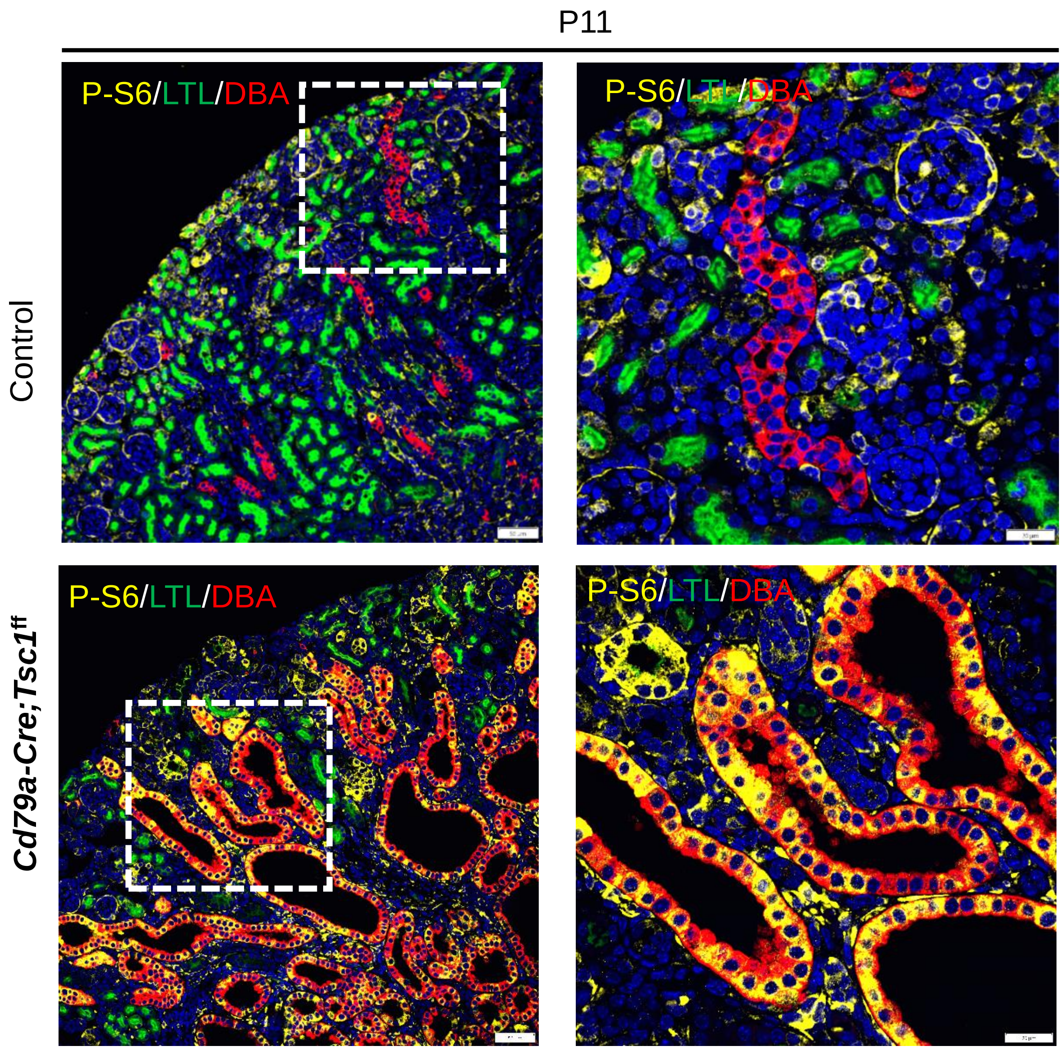

Supplementary Fig S9. Activation of mTOR pathways in Cd79a-Tsc1 KO kidney. Kidney sections at P11 are stained with phosphorylated-S6 (p-S6) antibodies (yellow) and markers for proximal tubules (LTL, green), and distal/collecting ducts (DBA, red). In control, p-S6-positive cells most frequently exist in proximal tubules in the superficial-cortex as well as cortico-medullary lesions as previously report (Zhou J, HMG, 2009; Centini R, PLoS One, 2018). By contrast, in Cd79a-Tsc1 KO kidneys, the cyst-lining cells in the distal/collecting duct stain more intensely for p-S6 signals. Note that not all of the cyst lining cells are positive for the p-S6. Dashed-box areas are magnified. Scale bars: x20 50µm, x60 20µm.

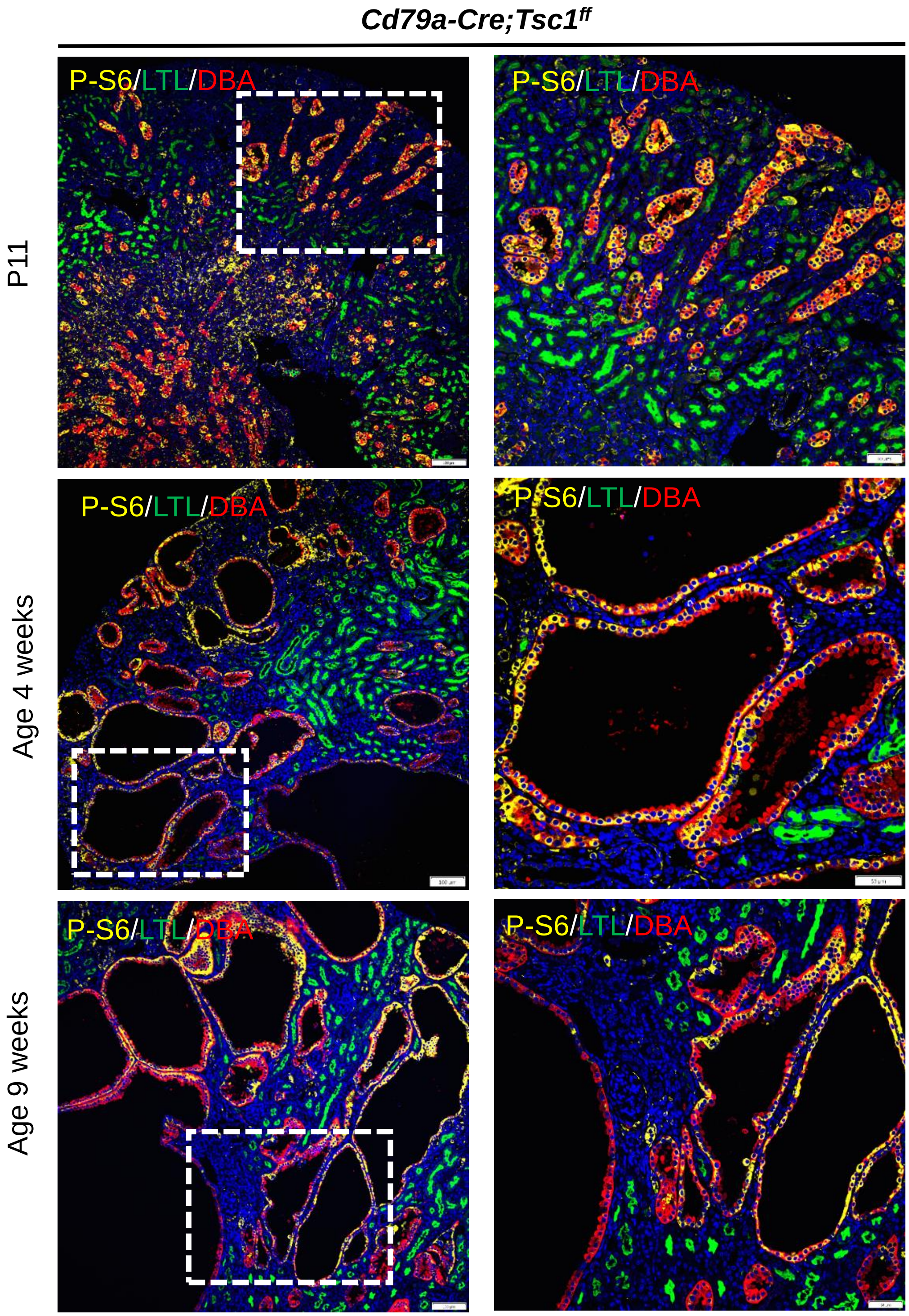

Supplementary Fig S10. P-S6 expression in tubules of Cd79-Tsc1 KO kidneys. Paraffin-embedded kidney sections of *Cd79a-Cre;Tsc1^{ff}* mice at P11, age of 4 weeks and 9 weeks are stained with p-S6 antibody (a downstream marker of mTORC1, yellow), LTL (proximal tubule, green), and DBA (distal tubule and collecting duct, red). Dashed-box areas are magnified.

a

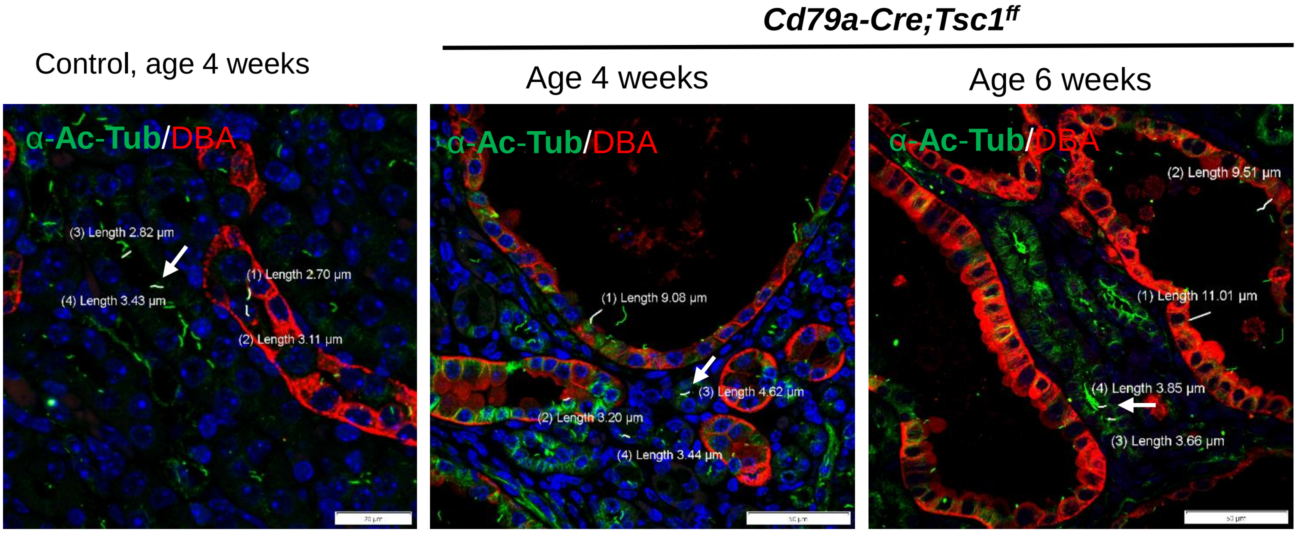

b

**Age**	**Tubular segment**	**Cilium length (μm, mean (SD))** Control	Cd79a-Tsc1 KO	Increase ratio *vs* control
9 days	Proximal tubules (DBA -negative)	3.093 (1.426) *n* =333	**3.839** (1.559) *n* =189	1.24
4 weeks		3.033 (1.032) *n* =580	**3.967** (1.212) *n* =400	1.31
6 weeks		3.194 (1.521) *n* =368	**3.798** (1.712) *n* =172	1.19
9 weeks		3.396 (1.260) *n* =639	**3.823** (1.660) *n* =222	1.13

Supplementary Fig S11. Cilia elongation in DBA-negative, proximal tubule cells.
(**a**) Kidney sections of Cd79a-Tsc1 KO at age 4 and 6 weeks and control are costained with anti-acetylated α-tubulin antibody (α-Ac-Tub, green), and DBA (red), the markers for cilia and the distal tubule/collecting duct, respectively. In Cd79a-Tsc1 KO tubules, cilia elongate in not only DBA-positive (distal to collecting duct) but also DBA-negative (proximal) segments (arrows), when compared with control. **(b)** Quantification of average cilia length over time during cyst development. Cilia elongation is observed for the proximal segments (DBA-negative), where mTOR is not activated by the Cd79a-Cre. The degree of lengthening is overall unchanged from early to late stage of cystogenesis. Such broader distribution of cilia elongation even in segments being remote from mTOR activation, suggesting the non-autonomous mechanisms. Data are indicated by means±SEM and are statistically analyzed by one-way ANOVA. Scale bars: 10μm, 20μm and 50μm.

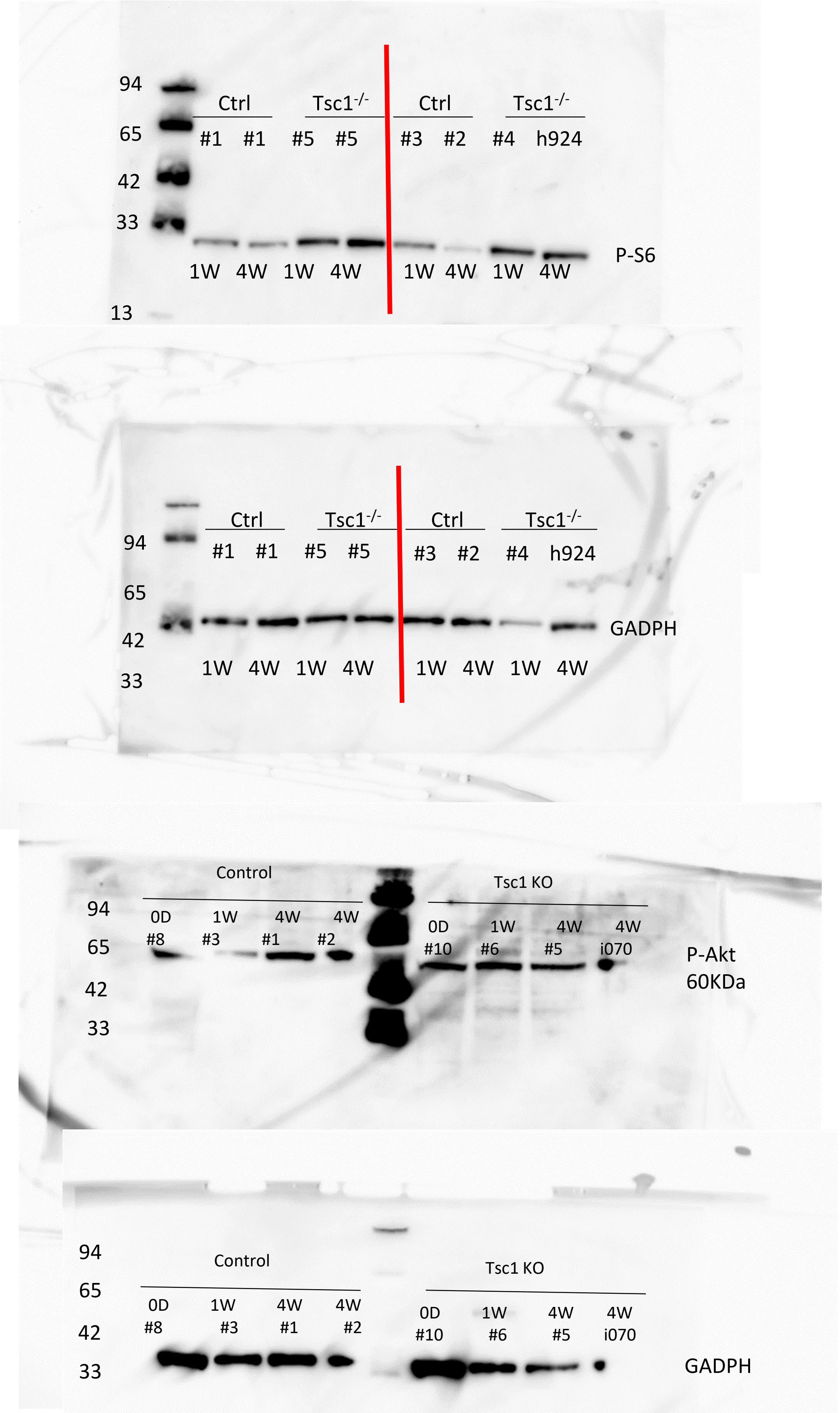

Supplementary Figure S12. The original images for cropped gel are presented in Figure 5.

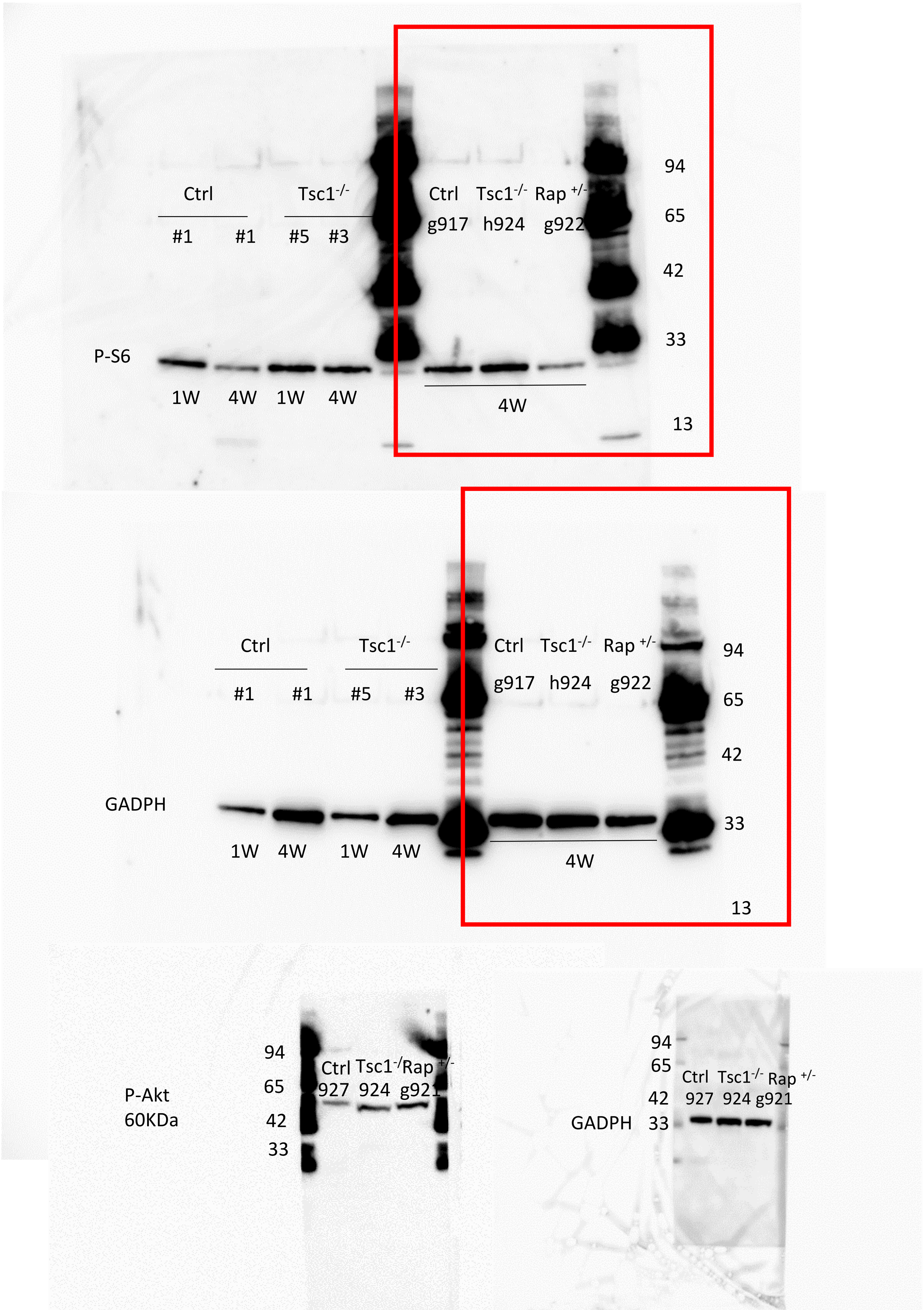

Supplementary Figure S13. The original images for cropped gel are presented in Figure 6.

Supplementary Table S1. List of Primary Antibody used in this study.

Antibody	Catalog number	Company	Dilution
DsRed (RFP)	632496	Takara	1:100
Wilms tumor protein (WT1)	SC-192	Santa Cruz	1:200
Dolichos biflorus agglutinin (DBA)	RL-1032	Vector	1:300
Lotus tetragonolobus lectin (LTL)	FL-1321	Vector	1:500
Aquaporin 2 (AQP2)	Sc-9882	Santa Cruz	1:200
Aquaporin 1 (AQP1)	ab9566	Abcam	1:200
Uromodulin	AF5144	Fujifilm	1:100
E-cadherin (E-cad)	610182	BD Bioscience	1:300
Phospho-histone H3 (pHH3)	9701S	Cell Signaling	1:200
Phospho-S6 Ribosomal Protein (P-S6)	D68F8	Cell Signaling	1:100
Ki-67	14-5698-82	Invitrogen	1:500
α-acetyl tubulin (α-Ac-Tub)	ab7291	Abcam	1:100
Proliferating cell nuclear antigen (PCNA)	NB500-106	Novus Biologicals	1:100

For immunofluorescence, paraffin sections (5mm) were first autoclaved at 115ºC for 20 minutes in citrate buffer pH 6.0 and were reacted with the first antibodies. All images were acquired using FV3000 Confocal Laser Scanning Microscope. For immunohistochemistry, paraffin sections (5mm) were treated with 3% hydrogen peroxide for 10 min. Antigen retrieval was done by microwave for 20 minutes in citrate buffer pH 6.0.

The mouse primary antibodies were visualized by Histofine® MOUSESTAIN and Simple Stain mouse MAX PO(M) kits (Nichirei Biosciences, 414322F, and 414341F, respectively).
The rabbit primary antibodies (PCNA, DsRed) were detected by Histofine® MOUSESTAIN and Simple Stain mouse MAX PO(R) kits (Nichirei Biosciences, 414322F, and 414341F, respectively).

PCNA- and TUNEL-positive nuclei were counted in randomly chosen area (0.05mm^2) of 5μm paraffin sections with intensive brown in the nuclei, whereas diffuse staining in the cytoplasm was excluded. Apoptosis was evaluated by TUNEL assay using ApopTag® Peroxidase In Situ Apoptosis Detection Kit (MERCK S7100).

Supplementary Table S2. Relationship between defective PCP and cystogenesis in the cilium-associated mutant models.

PCP signaling	Timing of action	Function	Genetic mode of action	Tubular cyst origin	Cilia length	Phenotype of tubular cells	References
CE	Predominantly in embryonic	Narrowing and elongation of tubules	*Kif3a*$^{cko/cko}$;*Ksp-Cre*	CD (P30)	Shortened	cell misorientation (E18.5)	Kunimoto K, Curr Biol, 2017
			Fat4$^{-/-}$	HL, CD (E16)	Shortened	cell misorientation in cochlea, aberrant OCD in kidney (P0)	Saburi S, Nat Genet, 2008
			Pkd1$^{\Delta C/\Delta C}$	All tubular segment (E15.5)	NA	cell misorientation (E15.5)	Castelli M, Nat Commun, 2013
OCD	Postnatal	Facilite longation of tubules	*Kif3a*$^{flox/-}$;*Pkhd1-Cre*	CD (P14)	Loss	aberrant OCD (P7-10)	Patel V,Hum Mol Genet, 2008
			Ift20$^{null/flox}$;*HoxB7-Cre*	CD (P10)	Loss	aberrant OCD (P5)	Jonassen JA, J Cell Biol, 2008
			Pkd1$^{flox/flox}$;*Mx1 Cre*	HL and CD (P1-2 mounths)	NA	aberrant OCD (P2-8 weeks)	Luyten A, J Am Soc Nephrol, 2010
			Tsc1$^{f/f}$;*Ksp-Cre*	CD (>P3 weeks)	NA	aberrant OCD (P20)	Bonucci M, Nature communicatios, 2020
			Tsc1$^{+/-}$, *Tsc2*$^{+/-}$, *Pkd1*$^{+/-}$	PT, HL and CD *(P9-12 months)*	Lengthened	aberrant OCD (P2-20)	Bonnet CS, Hum Mol Genet, 2009
			Pkhd1$^{del4/del4}$	no cyst develop	NA	aberrant OCD (P7-10)	Nishio S, J Am Soc Nephrol, 2010
			Pkd1$^{flox/-}$;*Ksp-Cre*	CD	NA	preserved OCD in precystic tubules (P3), aberrant OCD in dilated tubules (P3)	
			Pkd2$^{flox/flox}$;*Pkhd1-Cre;RA/EG*	CD (P8-10)	NA	preserved OCD in precystic tubules (P7), aberrant OCD in dilated tubules (P8-10)	

CD: Collecting duct; CE: Convergent extension; *Fat4*: Protocadherin Fat 4; HL: Henle's loop; *Ift*: Intraflagellar transport protein; *Kif*: Kinesin-like protein; NA: not available; OCD: Oriented cell division; PCP: Planar cell polarity; *Pkd*: Polycystin protein; PT: Proximal tubular; *Tsc*: Tuberous sclerosis.

Supplementary Table S3. Comparison of *Tsc1* versus *PKD1/2* inactivation model of mouse and human.

		This study	Mouse	Mouse	Mouse	Human
Gene		*Tsc1*	*Pkd1*	*Pkd1*	*Pkd2*	*PKD1 or PKD2*
Genetic mode of action		Conditional *Cd79a-Cre*	Conditional *γGt-Cre*	Germ-line B6Ola-*Pkd1*(*nl,nl*)	Germ-line *Pkd2*$^{WS25/WS25}$	Two hit one germ, one somatic
Tubular Cyst Origin	Early stage	DCT, CD in the cortex (P7-P14)	PT and DCT, CD (P3-P12)	PT in the cortex (P0-P7)	All tubular segment in the cortex and ourter medulla	Glomerulor cysts and all tubular segments
	Later stage	DCT, CD expansion to medulla (> 4 weeks)	DCT, CD > PT (> P12)	DCT, CD > PT expansion to medulla (> 2 weeks)	DCT, CD > PT	Predominant in CD with any segments
Early stage Cyst-lining Epithelia Morphology		Monolayer or multi-layer cuboidal and gradually becoming flattened	Monolayer of cells with cuboidal or flattened	Monolayer of cells with cuboidal or flattened	Monolayer of cells with cuboidal or flattened	Monolayer of cells with cuboidal or flattened
Proliferation		++ (> P14)	+ (> P2)	+ (> P4 weeks)	+	+
Apoptosis		+ (> P9 weeks)	+ (> P2)	+ (> P4 weeks)	+	+
Fibrosis		+ (> P9 weeks)	NA	+ (> P4 weeks) Regression	+ regression	+
Micropolyps and hyperplasia		+	NA	NA	+	+
References			Starremans PG, KI, 2008	Happe H, KI, 2013	Wu G, Cell, 1998	Grantham JJ, KI, 1987; Woo D, NEJM, 1995

CD: Collecting duct; DCT: Diatal convoluted tubule; HL: Henle's loop; NA: not available; PT: Proximal tubule; γGt: Glutamyl-transpeptidase.

Supplementary Table S4. Comparison of PKD phenotype due to Primary Cilia Defects vs mTOR activation.

PKD models	mTOR activation	Cilia defects				
Gene (Strain)	*Tsc1* KO	*IFT140*	*IFT*20	*Kif3A*	*Nek8 (jck)*	*NPHP3 (pcy)*
Mode of action	Conditional	Conditional	Conditional	Conditional	Germline *Nek8/Nphp9*	Germline *Nphp3*
Protein location		IFT complex A	IFT complex B	IFT motor	Cilia Inv compartment	Cilia Inv compartment
Function		Retrograde Transport Dynein motor	Antegrade Transport Kinesin-2 motor	Anterograde motor of cilia	Phosphorylates ANKS6, cilia signalling to cytoplasm	Modifier for ANKS6 phosphorylation
Promoter (timing switch-on)	Cd79a (E16) Ureteric bud	HoxB7 (E9 - E14) Mesonephric duct	HoxB7 (E9.5 - E12.5) Mesonephric duct and ureteric bud	Ksp (P1)	NA	NA
Tubular cyst origin	DCT and CD	CD	CD	CD(73%), DCT(8%), HL(5%)	DCT and CD	All tubular segments, predominantly in DT
Cyst onset	P7	P5	P10	P5	~P26	P1
Proliferation	Increase	Increase (> P5)	NA	Increase (P28)†	Increase (P50)†	Increase
Apoptosis	Increase (> P9 weeks)	Increase (> P5)	NA	Increase (P21)†	Increase (P50)†	NA
Cilia length	Lengthened	Shortened	Loss	Loss	Lengthened	NA
Orientation of cell division	Loss	Preseved	Loss	Loss	NA	NA
References		Jonassen JA,JASN, 2012	Jonassen JA, J Cell Biol, 2008	Lin F, PNAS, 2003; Patel V,Hum Mol Genet, 2008	Smith LA, JASN, 2006	Takahashi H, J Urol, 1986

Defects due to the primary cilia, either sensors (PKD1 or 2) or intraflagellar transport, converge on similar PKD phenotypes, with regards to the postnatal-onset, distal predominant, and proliferation characteristic of cysts.
ANKS6/Nphp16: Alpha motif domain containing 6; CD: Collecting duct; DCT: Distal convoluted tubule; E: Embryonic; HL: Henle's loop; IFT: Intraflagellar transport; NPHP: Nephronophthisis, P: Postnatal; PKD: .
†: a single point analysis only

Supplementary Table S5. Comparison of *Tsc1/2* inactivation model generated by various Cre-promoters.

Conditional	***Tsc1***				***Tsc2***		
Cre promoter	Cd79a	Nse	Ksp	Aqp2	*CAGGCre-ER^{TM}*	Aqp2	Abcg2-Cre^{ERT2}
Timing Cre recombination	~E16	E11.5-12.5	E14.5	Postnatal	TM (10 mg/kg) at E15.5	Postnatal	TM (0.5 mg/18g) at age 8-10 weeks
Tubular Cyst Origin	Ureteric bud	Mesenchymal	DCT and CD	Principle cell CD	Mesenchymal	Principle cell CD	PT
Cyst Onset	P7 days	P7 days	P20 days	P4 weeks	P6 weeks†	P5 weeks	P20 weeks
Cyst location	DCT and CD	PT, HL, and DCT	DT and CD	CD	NA	CD	PT
References		Zhou J, HMG, 2009	Bonucc M, Nat Commun, 2020	Chen Z, JASN, 2014	Liang N, J Exp Med, 2014	Kumar P, Genes Dis, 2022	Gewin LS, Am J Physiol Renal Physiol, 2019

Aqp2: Aquaporin 2; CD: Collecting duct; DCT: Distal convoluted tubule; E: Embryonic; HL: Henle’s loop; Ksp: Kidney-specific cadherin (Ksp-cadherin); NA: not available; *Nse*: neuron-specific enolase, P: Postnatal; PT: Proximal tubule; TM: Tamoxifen.

†: The onset of cystogenesis was unknown because of the single point observation.
